# Geography, phylogeny and host switch drive the coevolution of parasitic *Gyrodactylus* flatworms and their hosts

**DOI:** 10.1186/s13071-023-06111-6

**Published:** 2024-01-30

**Authors:** Hong-Peng Lei, Ivan Jakovlić, Shun Zhou, Xiang Liu, Chuan Yan, Xiao Jin, Bo Wang, Wen-Xiang Li, Gui-Tang Wang, Dong Zhang

**Affiliations:** 1 State Key Laboratory of Herbage Improvement and Grassland Agro-Ecosystems, and College of Ecology, Lanzhou University, Lanzhou, 730000 China; 2 Yangtze River Fisheries Research Institute, Chinese Academy of Fishery Sciences, Wuhan, 430223 China; 3 College of Fishery, Guangdong Provincial Key Laboratory of Aquatic Animal Disease Control and Healthy Culture, Guangdong Ocean University, Zhanjiang, China; 4 Shapotou Desert Research and Experimental Station, Northwest Institute of Eco-Environment and Resources, Chinese Academy of Sciences, 320 Donggang West Road, Lanzhou, 730000 People’s Republic of China; 5 Key Laboratory of Aquaculture Disease Control, Ministry of Agriculture, and Key Laboratory of Breeding Biotechnology and Sustainable Aquaculture, Institute of Hydrobiology, Chinese Academy of Sciences, Wuhan, People’s Republic of China

**Keywords:** Host-parasite interactions, Host specificity, Host use, Co-phylogeny, Coevolutionary events, Bipartite network

## Abstract

**Background:**

*Gyrodactylus* is a lineage of monogenean flatworm ectoparasites exhibiting many features that make them a suitable model to study the host-parasite coevolutionary dynamics. Previous coevolutionary studies of this lineage mainly relied on low-power datasets (a small number of samples and a single molecular marker) and (now) outdated algorithms.

**Methods:**

To investigate the coevolutionary relationship of gyrodactylids and their fish hosts in high resolution, we used complete mitogenomes (including two newly sequenced *Gyrodactylus* species), a large number of species in the single-gene dataset, and four different coevolutionary algorithms.

**Results:**

The overall coevolutionary fit between the parasites and hosts was consistently significant. Multiple indicators confirmed that gyrodactylids are generally highly host-specific parasites, but several species could parasitize either multiple (more than 5) or phylogenetically distant fish hosts. The molecular dating results indicated that gyrodactylids tend to evolve towards high host specificity. Speciation by host switch was identified as a more important speciation mode than co-speciation. Assuming that the ancestral host belonged to Cypriniformes, we inferred four major host switch events to non-Cypriniformes hosts (mostly Salmoniformes), all of which occurred deep in the evolutionary history. Despite their relative rarity, these events had strong macroevolutionary consequences for gyrodactylid diversity. For example, in our dataset, 57.28% of all studied gyrodactylids parasitized only non-Cypriniformes hosts, which implies that the evolutionary history of more than half of all included lineages could be traced back to these major host switch events. The geographical co-occurrence of fishes and gyrodactylids determined the host use by these gyrodactylids, and geography accounted for most of the phylogenetic signal in host use.

**Conclusions:**

Our findings suggest that the coevolution of *Gyrodactylus* flatworms and their hosts is largely driven by geography, phylogeny, and host switches.

**Graphical Abstract:**

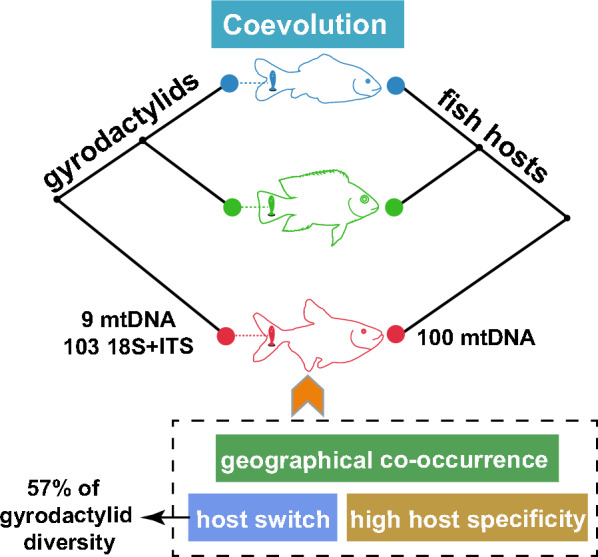

**Supplementary Information:**

The online version contains supplementary material available at 10.1186/s13071-023-06111-6.

## Background

Coevolution is the reciprocal evolutionary change in a set of interacting populations over time resulting from the interactions between those populations [1], and it is among the core topics in ecology and evolutionary biology [2]. Coevolution can be divided into two categories according to the relationship between species: mutualistic coevolution and antagonistic coevolution [3]. The relationship between parasites and their hosts belongs to the latter category: throughout the course of evolution, parasites and their hosts have been locked in a fierce evolutionary arms race, wherein they are forced to continually adapt to each other. Parasites are continuously optimizing their host invasion strategies, whereas their (potential) hosts are optimizing their evasion and defense strategies. This coevolution may result in co-speciation: the process wherein interacting groups, such as hosts and parasites, speciate in tandem [4]. In theory, this can generate congruent phylogenetic patterns between the two groups. However, in practice, phylogenetic congruence between hosts and parasites is usually either imperfect or absent due to a wide range of factors, comprising the incomplete lineage sampling, host switch, hybridization, and other evolutionary factors that result in incongruence between species phylogenies and the phylogenetic signal produced by individual genes [5]. As a result, phylogenetic congruence is difficult to identify with confidence. For example, Desdevises et al. [6] conducted a study on the associations between a group of parasites (*Lamellodiscus spp.*) and their hosts and concluded that there was no significant signal of co-speciation and specialization; instead, they proposed that host-parasite associations are driven primarily by ecological factors. Similarly, Vienne et al. [7] showed that convincing cases of co-speciation were rare (7%) and that co-phylogenetic methods overestimated the occurrence of such events.

Host specificity denotes the number of host species that a parasite infects in its natural habitat, so it is commonly used as a measure of a parasite's potential to switch between different host species [8]. It is also considered to be the key parameter that determines the complex coevolutionary relationship between parasites and hosts, and the process of species formation [9]. In general, the stronger the host specificity (defined as the level of genetic adaptation to a specific host), the better it should reflect the internal mechanism of the coevolution of parasite and host [10]. Two parasites that infect the same number of host species nominally have identical host specificities. However, their real host specificity can differ at numerous levels when the geography and phylogeny of hosts are taken into consideration, so there are multiple ways to quantify host specificity. Aside from the number of host species infected by a parasite, scientists have also recognized phylogenetic, geographic, and phylobeta host specificity (combining phylogenetic and geographic specificity) [11]. Phylogenetic host specificity is estimated using the standardized effect size of phylogenetic specificity (SPS_i_) parameter, and phylogenetic specificity represents the total length of branches linking the host species of parasite *i* along the phylogenetic tree [11]. Geographic host specificity is estimated using the BS_i_ parameter, which estimates the dissimilarity in host species identities across localities [11]. Phylogenetic host specificity and geographic host specificity can be combined into a single index, PBS_i_, which represents the phylogenetic distance among host species assemblages used by a parasite *i* over a geographic space [11].

Due to their comparatively high host specificity [12], as well as their direct life cycle (they have no intermediate hosts), “monogeneans” are an important parasitic model for the research of host-parasite coevolution [13]. *Gyrodactylus* is a speciose monogenean (Gyrodactylidea: Gyrodactylidae) genus comprising around 400—500 species [14, 15]. Their distribution is global, they encompass both highly host-specific and generalist species, and they infect a broad range of hosts [14]. *Gyrodactylus* species have a high breeding rate and a short generation time, and they are unique among monogeneans for being the only lineage that reproduces viviparously [12]. They are also capable of surviving for some time independently of their host, and basic swimming capabilities have been observed in some species [16]. Some of their characteristics, in particular hyperviviparity, parthenogenesis, and the ability of transmission between hosts in adult parasites, facilitate high population growth rates and high colonizing capabilities of gyrodactylids [17–20]. In addition, they are easy to cultivate on the body surface of the host, and they have been maintained in pure lineage culture systems for > 10 years [12], which also makes them a good model for evolutionary studies.

Coevolutionary relationships between gyrodactylids and their hosts were studied before. Huyse et al. [21] conducted co-speciation phylogenetic analyses using the V4 region of the *18S rRNA* and the complete *ITS rDNA* region for the gyrodactylids and *12S* and *16S* mtDNA fragments for the hosts. They found that the inference of coevolutionary relationships between gyrodactylids and hosts was strongly affected by the software algorithm used: algorithms based on the topological structure found significant signals of coevolution, but algorithms based on distance methods did not find significant coevolutionary relationships. Hahn et al. [22] conducted co-speciation analyses using mitochondrial *cox1* and nuclear ribosomal *ITS2*. They found significant evidence for global congruence between host and parasite phylogenies and for co-speciation between *Gyrodactylus teuchis* and its salmonid hosts *Salmo trutta* and *Salmo salari* using distance-based algorithms (Parafit and PACo). However, both of the above studies relied on limited species sampling and markers with poor resolution (morphological characters and relatively small DNA sequences). No (mito)genome-wide studies of gyrodactylid host-parasite co-speciation have been conducted so far.

In this study, we revisited the coevolution of gyrodactylids and their hosts using two datasets for gyrodactylids and hosts. As we identified only seven *Gyrodactylus* mitogenomes suitable for these analyses, to increase the resolution, we sequenced two new mitogenomes: *Gyrodactylus* sp. L1 and *Gyrodactylus* sp. L4 (Additional file 1: Figure S1). Using the above datasets, we comprehensively studied the coevolution of gyrodactylids and their hosts using four commonly used software programs, Treemap 3, ParaFit, PACo, and Jane 4. We used two different sub-datasets to test the hypotheses that host species infected by multiple parasite species could cause incongruence between the host and parasite phylogenies [23] and that low host specificity of parasites may lead to inconsistent phylogenetic processes [24]. We also analyzed the role of host switches in the reconstruction of the coevolutionary history and quantified the impact of major host switches on the species diversity in gyrodactylids. This also allowed us to explore the effect of host specificity and host switch on the coevolution of gyrodactylids and their fish hosts. Finally, we used the network analysis to analyze the dataset of 103 gyrodactylid species parasitizing 100 different host species; in one network we depicted the interactions between gyrodactylids and their hosts, and in the other network we characterized the global co-occurrence of gyrodactylids. As utilization of host resources is a key indicator of coevolution between parasites and the hosts, we also studied the host use by gyrodactylids in both phylogenetic and geographical space using the community detection analysis (module analysis).

## Methods

### Host and parasite data

Both sequenced *Gyrodactylus* parasites were collected from *Schizopygopsis pylzovi* (Cyprinidae) caught in October 2021 in the Yellow River, Lanzhou, China (sampling and identification details in Additional file 2)*.*

Co-phylogenetic analyses were conducted on two species-for-species matching host and parasite datasets: (i) PHMITOS—the mitogenome dataset for nine parasites and nine hosts; (ii) P18SHMITO—*18S rRNA* + *ITS* for *Gyrodactylus* parasites (103 species) and mitogenomes of 100 host species. Seventeen *Gyrodactylus* mitogenomes were available in the GenBank database (last accessed 9 June 2022). After removing duplicates (leaving one mitogenome per species) and two mitogenomes for which we failed to find the host information, seven mitogenomes were left in the dataset; to this we added two newly sequenced and annotated mitogenomes, resulting in nine mitogenomes in total. Similarly, 378 *Gyrodactylus* 18S + *ITS* gene sequences were downloaded from GenBank (last accessed 9 June 2022), and 103 sequences were left after the same filtering procedure. The host information for the above two parasite datasets was obtained from the GyroDb database [25]. We also built two additional P18SHMITO subdatasets for additional analyses. (i) Among the 100 hosts in our study, only 17 were parasitized by more than three gyrodactylids. To assess their impact on the analyses, we removed them, as well as their unique parasites, from the P18SHMITO dataset. This dataset, named “17 hosts removed,” comprised 82 gyrodactylids and 83 hosts. (ii) To test whether gyrodactylids with low host-specificity interfered with the analyses, we removed gyrodactylids with the top 15% SPS_i_ values (see the definition of SPS_i_ in the “Host specificity” section), and their unique hosts, from the P18SHMITO dataset. This subdataset was named “removed top 15% SPS_i_,” and it comprised 88 gyrodactylids and 85 hosts.

### Phylogenetic analyses

PhyloSuite v.1.2.3 [26, 27] was used to retrieve the data from GenBank, extract and modify the default taxonomic data, extract mitogenomic data, parse and extract the mitogenome annotations recorded in Word documents, create GenBank submission files, create organization tables for mitogenomes, make genomic statistics of the mitogenomes of gyrodactylids, generate annotation files for the architecture visualisation in iTOL [28], and conduct phylogenetic analyses using two plug-in software programs: IQ-TREE (maximum likelihood analysis—ML) [29] and MrBayes (Bayesian Inference—BI) [30]. Two datasets were used: (i) nucleotide alignment of all PCGs + 2rRNAs of the PHMITO dataset; (ii) nucleotide alignments of *18S* + *ITS* (gyrodactylids) and 13PCGs + 2rRNAs (hosts) of the P18SHMITO dataset. All preparatory steps were also conducted using PhyloSuite and its plug-in programs: sequences were aligned in MAFFT [31] and PCG alignments further refined using MACSE [32]; alignments were concatenated by PhyloSuite, and optimal evolutionary model and partitioning strategy were inferred using ModelFinder (for *18S* + *ITS* data) [33] and PartitionFinder2 (for mitogenome data) [34].

### Host specificity

For each gyrodactylid species, we calculated its basic host specificity, phylogenetic host specificity, geographic host specificity, as well as phylobeta host specificity. Basic host specificity was calculated with the number of recorded host species or ‘host species range.’ Phylogenetic host specificity (SPS_i_) was represented by the standardized effect size of Faith’s phylogenetic diversity (PD) of hosts exploited by a parasite, calculated using the R function ses.pd in the Picante package [35]. The standardized effect size of phylogenetic specificity (SPS_i_) was inferred as the difference between the observed phylogenetic specificity (PS) between hosts of each gyrodactylid and the mean phylogenetic specificity ($\overline{{{\text{PS}} }_{{\text{sim}}}}$) obtained with the same number of hosts generated using a random choice of host species from the tree (null data), divided by the standard deviation of phylogenetic specificity in the null data. PS_sim_ values were averaged over 999 runs and 1000 iterations, as recommended by Poulin, Krasnov and Mouillot [11].

$$SP{S}_{i}=\frac{\left(P{S}_{i}-\overline{P{S }_{sim}}\right)}{SD\left(P{S}_{sim}\right)}$$

Phylogenetic distance linking the host species could not be inferred in cases of gyrodactylids with a single reported host. As they should have the lowest phylogenetic specificity value, we manually set the SPS_i_ value of parasites with only one host to correspond to the lowest SPS_i_ value observed in our results (–4.7).

Geographic host specificity (BS_i_) was calculated to estimate the dissimilarity in host species identities across localities. The geographic distribution range of hosts was obtained from FishBase [36]. Data limitations currently prevent us from establishing a global geographic distribution map for gyrodactylids. In a highly simplified model, we would expect the geographic distribution map of gyrodactylids to match that of their hosts. In parasite-host interactions, local parasite species richness is often driven by local host richness in a bottom-up fashion [37]. On regional scales, numerous studies have consistently demonstrated a positive correlation between host richness and parasite richness in extensively sampled local habitat patches [38–40]. A meta-analysis has further validated the robustness of this relationship, observing its strong presence across diverse taxa of hosts and parasites [41]. Based on the assumption that this pattern holds true at larger spatial scales, supported in a previous study [42], for the purpose of this study we predicted that the global distribution of parasite biodiversity, for any specific group of parasites and level of host specificity, may broadly mirror that of their hosts. We also discuss the limitations of this assumption (see Additional file 2: Limitations). We used an extension of the Sørensen dissimilarity index for multiple sites (BS_i_) to measure the geographic host specificity:

$$B{S}_{i}=1-\frac{T}{T-1}\left(1-\frac{{S}_{T}}{{\sum }_{t} {S}_{t}}\right)$$

where T is the number of samples or localities, S_t_ is the number of host species used by the parasite on the locality t, and S_T_ is the total number of host species used by the parasite species *i* across all T localities (i.e. the regional host pool). If parasite *i* exploits the same host species across all localities, then S_t_ = S_T_ and BS_i_ = 0. If a parasite *i* uses completely different host species at different localities, then S_T_ = ${\sum }_{t} {S}_{t}$ and BS_i_ = 1.

For the phylobeta host specificity, we used an extension of the Sørensen index to branches instead of species following the principle underlying the construction of the Phylosor index.

$$PB{S}_{i}=1-\frac{T}{T-1}\left(1-\frac{P{D}_{T}}{{\sum }_{t} P{D}_{t}}\right)$$

where T is the number of samples or localities, PD_t_ is the phylogenetic diversity of host species used by the parasite at locality t, and PD_T_ is the phylogenetic diversity of all host species used by the parasite species *i* across all T localities. If a parasite *i* exploits the same host species across all localities, then PD_t_ = PD_T_ and PBS_i_ = 0. If a parasite *i* uses different host species at different localities, then the PBS_i_ value is inversely correlated to the phylogenetic relatedness of hosts (the less phylogenetically related the hosts are, the higher the PBS_i_ value).

### Testing for cospeciation

We used four methods to test the co-speciation for gyrodactylids and their hosts. Two of these were topology-based methods: TreeMap 3 [43] and Jane 4 [44], as this type of method can be negatively affected by the presence of phylogenetic artifacts in host or parasite topologies [21]. To assess the reliability of the results of these two algorithms, we also used two additional algorithms, which make use of raw or patristic distances: ParaFit [45] and PACo [46].

Topology-based methods:

TreeMap 3 was designed to test for statistically significant topological congruence between two given phylogenies, which in turn tests for the hypothesis of a history of co-divergence between mimetic populations.

Jane 4 is an algorithm for co-phylogeny reconstruction. The input file for Jane 4 is also a pair of phylogenetic trees (the host and parasite trees) and a map recording the associations between parasites and hosts. Jane 4 reconciles the host and the parasite tree by introducing five types of events: co-speciation, duplication, duplication & host switch, loss, and failure to diverge events. Each of these five event types has an associated cost that may be specified by the user, and the Jane 4 algorithm seeks to find a mapping with the minimal total cost. The algorithm was run with a population size of 500 for 20 generations using the default costs of three types of events: co-speciation = 0, duplication = 1, and failure to diverge = 1. Based on the characteristics of gyrodactylids (see Introduction) and referring to Hamerlinck et al. [47], we slightly adjusted the costs of two types of events: duplication & host switch = 1 (default = 2), and loss = 2 (default = 1). In more detail, as gyrodactylids can easily detach themselves from hosts and spread between hosts via contact transmission, we estimated that the cost of host switch should be less than that of duplication and the cost of loss should be the highest. The significance of the co-phylogenetic signal was estimated by re-running the algorithm on 100 randomly permuted host-parasite associations with the same settings and comparing the resulting cost distribution with the observed cost.

Distance-based methods:

ParaFit and PACo programs statistically assess the fit between the host and parasite phylogenetic distance matrices mediated by the matrix of host-parasite links. As input data for the two software programs, we inferred genetic distances from the host and parasite phylogenetic trees using the cophenetic.phylo function in the ape package in R [48] and then converted them to matrices.

### Phylogenetic patterns in host switch

We compiled all pairs of fish species connected by a gyrodactylid (e.g. if a gyrodactylid had four host species, six fish pairs were recorded). Then, we calculated the phylogenetic distance between each fish species pair using pairwise patristic distance analysis implemented in the TreeSuite function in PhyloSuite and arranged the phylogenetic distances of fish pairs into 10 distance bins. Finally, we calculated the proportion of fish pairs falling into each of the 10 phylogenetic distance bins. In this way, we calculated the probability that a fish pair sharing a gyrodactylid has a given phylogenetic distance.

### Evaluation of the divergence time of gyrodactylids

To gain a more comprehensive understanding of the evolutionary history of gyrodactylids and the evolutionary trends of their host specificity, we evaluate the divergence times of gyrodactylids. The topology obtained using IQ-TREE and P18SHMITO dataset was used for a subsequent dating analysis performed using the MCMCTREE tool of the PAML 4.9 package [49]. We used two calibration points for the inference of divergence time in gyrodactylids. According to the available data, the differentiation time of the most common ancestor of *Gyrodactylus vimbi* and *G. teuchis* was set between 0.8 and 2.2 Mya [22], and the differentiation time of the most common ancestor of *G. pannonicus, G. albolacustris, G, danastriae*, and *G. botnicus* was set between 1.3 and 1.8 Mya [50]. Burnin was set to 400,000 (400 K), sampfreq to 10, and nsample to 100 K. The convergence of MCMCTREE runs was checked using the program Tracer [51].

### Correlation analysis

To compare the relationships between different host specificities, as well as the variation of host specificity throughout evolutionary history, and the contribution of host specificity to the consistency of co-speciation events, we conducted correlation analyses for four kinds of host specificity, divergence time, and *p* values of host-parasite individual links in Parafit. We performed correlation analysis using the Pearson correlation coefficient to assess the relationships between the variables mentioned earlier.

### Network analysis

To map the host use by gyrodactylids, we used the community detection analysis, borrowed from network theory, to study network topological communities or modules [52]. Community detection approaches have been applied previously to explore the compartmentalization of occurrence networks [53, 54]. First, we created a bipartite interaction network based on the known interactions between gyrodactylid parasites and their hosts. Bipartite networks are composed of two subsets of nodes, or levels (for instance hosts and parasites), where links occur only between nodes of different levels. In contrast to the typical applications of interaction networks that focus on studying co-occurring species within a community, our approach focused on constructing a global network that encompasses the documented interactions between gyrodactylid parasites and their fish hosts, regardless of their co-occurrence in specific locations. We created a spatial co-occurrence network (geographic network) with a bipartite structure: gyrodactylids and the regions where they occur constituted two different subsets of nodes, establishing a link based on the presence of a given species at a given locality.

When applied to a bipartite network, community detection analysis informs which groups of nodes from both levels are more densely connected. In the interaction network, this refers to groups of hosts and their associated gyrodactylids, whereas in the geographic network, this refers to groups of gyrodactylids occurring in the same regions. To analyze the modular structure of both networks, we used the modularity index proposed by Barber [55] for bipartite networks. This index was optimized using the Louvain algorithm [56, 57], and it was implemented in the function “Gen Louvain” [58] in MATLAB. Finally, to test whether our networks were more modular than expected for a random network, we compared the observed values of modularity against the distributions of 100 random networks. We then calculated the proportion of random networks that were equally or more modular than the observed network. To generate the null models we used the independent swap algorithm implemented in the R package picante [59]. This algorithm keeps a constant node degree (i.e. row and columns marginal total) as well as network size and connectivity.

### Phylogenetic structure of gyrodactylid-host modules

To investigate the phylogenetic structure within modules, we calculated the extent to which taxa belonging to a given module were, on average, more closely related within them than with taxa from other modules. In other words, we conducted calculations for the phylogenetic mean pairwise distances (MPD) between the taxon *i* and all other taxa within the same module (MPD_i intracommunity_) and subtracted the mean pairwise distances of the taxon *i* and all other taxa from different modules (MPD_i intercommunity_). Subsequently, we determined the relative phylogenetic distinctiveness (RPD) by taking the reciprocal of the average value among all taxa within a module, resulting in higher values when the phylogenetic distinctiveness was greater:

$$RPD=-1*\frac{{\sum }_{i=1}^{N} MP{D}_{i\text{ intracommunity }}-MP{D}_{i\text{ intercommunity }}}{N}$$

N is the number of species in a given module. To test whether gyrodactylids belonging to a module were more phylogenetically distinct than a random array of lineages, we recalculated this index 9999 times by randomizing the tip labels of the gyrodactylid phylogeny. The probability [60] of being phylogenetically distinctive was then calculated as the proportion of these 9999 null cases being more or equally distinctive than the observed phylogeny. We calculated the proportion of significant (*p* ≤ 0.01) cases for each module.

### Effects of geographical co-occurrence on the phylogenetic structure of host use

We also explored to what extent the geographic distribution of gyrodactylids accounts for the phylogenetic structure of interaction modules. To do so, we calculated the degree to which gyrodactylids belonging to the same interaction module were, on average, more closely related among themselves than with the species occurring in the same geographical module. By modifying the RPD index, we calculated MPD_intracommunity_ for each gyrodactylid of a given interaction module in the same way as previously explained. However, when calculating the MPD_intercommunity_, we only considered the gyrodactylids that co-occur in the same geographical module. The remaining calculations were done as explained above for the RPD index. Finally, significance was assessed following the above-explained randomization procedure.

### The relative weight of phylogeny and geography in shaping host use in gyrodactylids

To measure both host use and geographic dissimilarities, we used the Simpson dissimilarity index [61] as implemented in the R package betapart [59]. We then fitted a generalized linear regression to host use dissimilarities as a response to geographical dissimilarities and phylogenetic distances. Furthermore, to identify significant relationships, we randomized the matrix 500 times using the independent swap algorithm as implemented in picante [35] to get 500 null cases according to the method of Calatayud et al. [62]. We calculated host use dissimilarities for these 500 null cases and interpreted coefficients > 95% of the null cases as significant.

## Results

### Host specificity

**Fig. 1:**
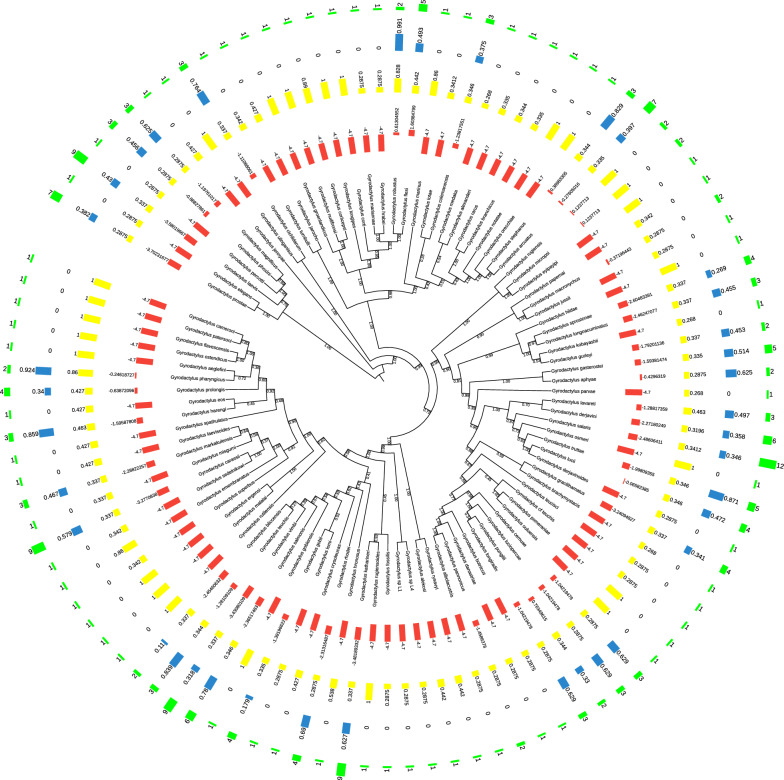
Phylogeny of 103 gyrodactylids inferred using *18S rRNA* and *ITS* genes and the Maximum Likelihood methodology implemented in IQ-TREE. The clade comprising six *Gyrodactylus* species (*G. laevis, pecotti, magnificus, phoxini, elegans*, and *prostae*) was used as the outgroup. The figure shows (from inside to outside): the tree of gyrodactylids with bootstrap values, phylogenetic host specificity, phylobeta host specificity, geographic host specificity, and basic host specificity bar

Many gyrodactylid species in our dataset were recorded from one species of host, but several species were recorded from a large number of hosts (up to 12) (Fig. 1). The average SPS_i_ value of gyrodactylids was – 3.395, and the vast majority (94.2%) exhibited SPS_i_ values < 0. The BS_i_ value ranged from 0.268 to 1, with an average of 0.516. Only gyrodactylids sampled at one locality had a BS_i_ value of 1. The PBS_i_ value ranged from 0 to 0.991, with an average of 0.193, but a majority (64%) of gyrodactylids exhibited PBS_i_ = 0.

### Evidence for coevolution

#### Treemap 3 (topology-based method)

Using TreeMap 3, we obtained a coevolutionary scenario that represents the best way to associate host and parasite phylogenies and statistically test the significance of co-phylogenetic reconstruction. Tanglegrams produced by Treemap 3 comprised a host tree, a parasite tree and a set of associations (the host range of each parasite) (Additional file 1: Figures S9–S12). There was a significant congruence between two trees (phylogenetic correlation between parasites and hosts was significant) in ML and BI tanglegrams of the PHMITOS dataset: four nodes exhibited significant congruence between host and parasite subtrees in the parasite tree and three/four nodes in the host tree of ML/BI tanglegrams (Additional file 1: Figures S9 and S10; significant subtree nodes are indicated by red dots). Tanglegrams of ML and BI trees of the P18SHMITO dataset were complicated, and associations were difficult to distinguish (Additional file 1: Figures S11 and S12). Compared with the PHMITOS dataset, the number of congruent nodes in the host tree increased (0.415 per host vs. 0.385 per host, Table 1), but the number of significantly congruent nodes in the parasite tree decreased (0.192 per parasite vs. 0.440 per parasite, Table 1). The significance of congruence was also higher in the P18SHMITO dataset than in the PHMITOS dataset. After removing 17 hosts parasitized by more than three gyrodactylids, the significance of congruence between gyrodactylid and host topologies decreased strongly (0.169 significantly congruent nodes per host and 0.159 per parasite, Table 1) compared with the results of P18SHMITO dataset (0.415 significantly congruent nodes per host and 0.192 per gyrodactylid). After eliminating the top 15% gyrodactylids with the highest SPS_i_ values, we observed a slightly decreased significance of congruence between gyrodactylids and hosts (0.376 significantly congruent nodes per host and 0.159 per gyrodactylid, Table 1) compared with the results of the P18SHMITO dataset (0.415 significantly congruent nodes per host and 0.192 per gyrodactylid).

**Table 1:** Number of nodes exhibiting significant congruence in Treemap 3 results

Dataset	SN per host	SN per parasite
PHMITOS-ML	0.330	0.440
PHMITOS-BI	0.440	0.440
P18SHMITO-ML	0.410	0.184
P18SHMITO-BI	0.420	0.200
17 hosts removed	0.169	0.159
removed top 15% SPS_i_	0.376	0.159

*SN*  nodes with significant congruence between the two topologies

#### ParaFit and PACo (distance-based methods)

We conducted distance-based analyses in ParaFit and PACo using PHMITOS ML and BI trees. ParaFit analysis produced two statistics for each of the 11 host-parasite links, which identified the links that significantly contributed to the co-phylogenetic pattern observed. Both analyses revealed a significant global co-phylogenetic structure (Table 2). These results were confirmed using PACo analysis, which also found evidence for a significant global congruence between the host and parasite in the PHMITOS dataset. ParaFit also found that six out of 11 individual host-parasite links contributed significantly to the overall co-phylogenetic structure in both analyses. We also conducted the same set of analyses using P18SHMITO ML and BI trees. ParaFit and PACo analyses both revealed a highly significant global co-phylogenetic structure. ParaFit indicated that out of 234 individual host-parasite links, 65.38% contributed significantly to the overall co-phylogenetic structure in the ML topology and 64.96% in the BI topology.

**Table 2:** ParaFit and PACo results for all datasets

Dataset	ParaFit	Links	PACo
PHMITOS-ML	0.030	6 (total 11)	0.007
PHMITOS-BI	0.023	6 (total 11)	0.009
P18SHMITO-ML	0.001	153 (total 234)	< 0.001
P18SHMITO-BI	0.001	152 (total 234)	< 0.001
17 hosts removed	0.001	90 (total 122)	< 0.001
removed top 15% SPS_i_	0.001	128 (total 187)	< 0.001

The ParaFit column lists the *p* values of the ParaFit result. The links column lists the number of host-parasite individual links that contributed significantly to the overall co-phylogenetic structure in ParaFit. The PACo column lists the *p* values of the PACo result

In the “17 hosts removed” dataset, the global congruence between the host and parasite phylogenies was highly significant (*p* = 0.001 in ParaFit and *p* < 0.001 in PACo), and the percentage of individual host-parasite links that contributed significantly to the overall co-phylogenetic structure increased to 73.77%. Global congruence between the host and parasite phylogenies was also highly significant after eliminating the top 15% of gyrodactylids with the highest SPS_i_ values (*p* = 0.001 in ParaFit and *p* < 0.001 in PACo).

#### Jane 4 (topology-based method)

We used the Jane 4 algorithm and different trees (Additional file 1: Figures S2–S8) to infer the following types of events: co-speciation, duplication, duplication & host switch, loss, and failure to diverge (Table 3; Additional file 1: Figures S13–S16). First, we tested the cost setting of 01121, where the numbers represent the costs of the following parameters from left to right: co-speciation, duplication, duplication & host switch, loss, and failure to diverge. Under this setting, the two PHMITOS dataset analyses (ML and BI) produced identical results, with the number of duplication & host switch events greater than the number of co-speciation and a total cost of events of 12. We further tested the event cost setting of 01322, wherein we greatly increased the cost of duplication and & host switch events to 3. Despite these changes, the number of co-speciation events did not exceed the number of duplication & host switch events, while the total cost of events increased. Using the original cost setting (01121), the two P18SHMITO dataset trees produced different results, with the ML tree producing a somewhat higher total cost of events (1324) than the BI tree (1302), but the numbers of duplication & host switch events were greater than the number of co-speciation events. When P18SHMITO analyses were run with the cost setting of 01322, the results were similar (Table 3). The Jane 4 results showed that the fractions of the loss and failure to diverge events per parasite were strongly reduced in the “17 hosts removed” dataset (1.817 vs. 5.311 and 0.500 vs. 1.291 respectively). Also, the total cost was reduced from the average of 12.854 per parasite in the P18SHMITO dataset to 5.037 per parasite (413 in total). In the “removed top 15% SPS_i_” dataset, the number of the loss events per parasite was strongly reduced (3.250 vs. 5.311), whereas the number of failures to diverge events changed only slightly (1.136 vs. 1.291 respectively). The average total cost per parasite was also reduced to 8.545 per parasite (a total of 752) compared to the P18SHMITO dataset (12.854).

**Table 3:** Number of events and associated cost for all datasets inferred using the Jane 4 algorithm

	Event costs	Co-speciation	Duplication	Duplication & Host Switch	Loss	Failure to diverge	Total cost	No. of parasites
PHMITOS-ML	01322	3	2	3	3	2	21	9
PHMITOS-ML	01121	2	1	5	2	2	12	9
PHMITOS-BI	01322	1	2	5	3	2	27	9
PHMITOS-BI	01121	2	1	5	2	2	12	9
P18SHMITO-ML	01322	8	37	57	551	133	1576	103
P18SHMITO-ML	01121	5(0.049)	25(0.243)	72(0.699)	547(5.311)	133(1.291)	1324(12.854)	103
P18SHMITO-BI	01322	9	37	56	545	133	1561	103
P18SHMITO-BI	01121	7	32	63	537	133	1302	103
17 hosts removed	01121	7(0.085)	20(0.244)	54(0.659)	149(1.817)	41(0.500)	413(5.037)	82
removed top 15% SPS_i_	01121	7(0.080)	20(0.227)	60(0.682)	286(3.250)	100(1.136)	752(8.545)	88

The numbers in brackets represent the number of events divided by the corresponding number of parasites

### Phylogenetic patterns in host switch

Most gyrodactylids in our dataset tended to parasitize on phylogenetically close hosts, with 74.94% of host pairs exhibiting a phylogenetic distance < 0.8 (Additional file 1: Figure S17, panel A). The probability that two closely related fish species (patristic distance ≤ 0.5) shared the same gyrodactylid was high (> 50%; Additional file 1: Figure S17, panel A), while the probability that more distantly related fish species (patristic distance ≥ 1) shared the same gyrodactylid was much lower (≤ 23%). The fish pairs here mainly comprised fish species with multiple gyrodactylid parasites (Additional file 3: table S1). Furthermore, phylogenetic patterns of parasite pairs that shared the same fish host exhibited a bimodal distribution (Additional file 1: Figure S17, panel B and Additional file 3: Table S2).

### Comparison of host specificity

We conducted a correlation analysis of the number of host species of gyrodactylids and the SPS_i_ value. After removing gyrodactylids with only one host, the number of host species was significantly negatively associated (correlation coefficient = – 0.58 and *p* value = 7.7E–05) with the SPS_i_ value (Additional file 1: Figure S18), whereas the two parameters were positively correlated when the full dataset was used (correlation coefficient = 0.45 and *p* value = 1.52E–06). We conducted a nonparametric test to assess whether there were differences in SPS_i_ values between gyrodactylids in the last five patristic distance bins and those in the first two patristic distance bins: although both groups had negative average SPS_i_ values (– 0.41 and − 3.83 respectively), values in the last five bins group were significantly lower (Mann-Whitney U test = 1103, *p* < 0.001). Moreover, we conducted a nonparametric test to verify whether there were differences in SPS_i_ values between parasites that significantly contributed to the co-phylogenetic pattern in Parafit results and those that did not. The result showed that gyrodactylids that significantly contributed to the co-phylogenetic pattern had significantly lower SPS_i_ values than the others (Mann-Whitney U test = 660, *p* < 0.001), but the average values of the two groups were both negative (− 3.97 and − 2.52 respectively). We finally conducted a correlation analysis of the divergence time of gyrodactylids and the SPS_i_ value: the correlation coefficient was − 0.23 (*p* value = 0.02).

### Network analysis

**Fig. 2:**
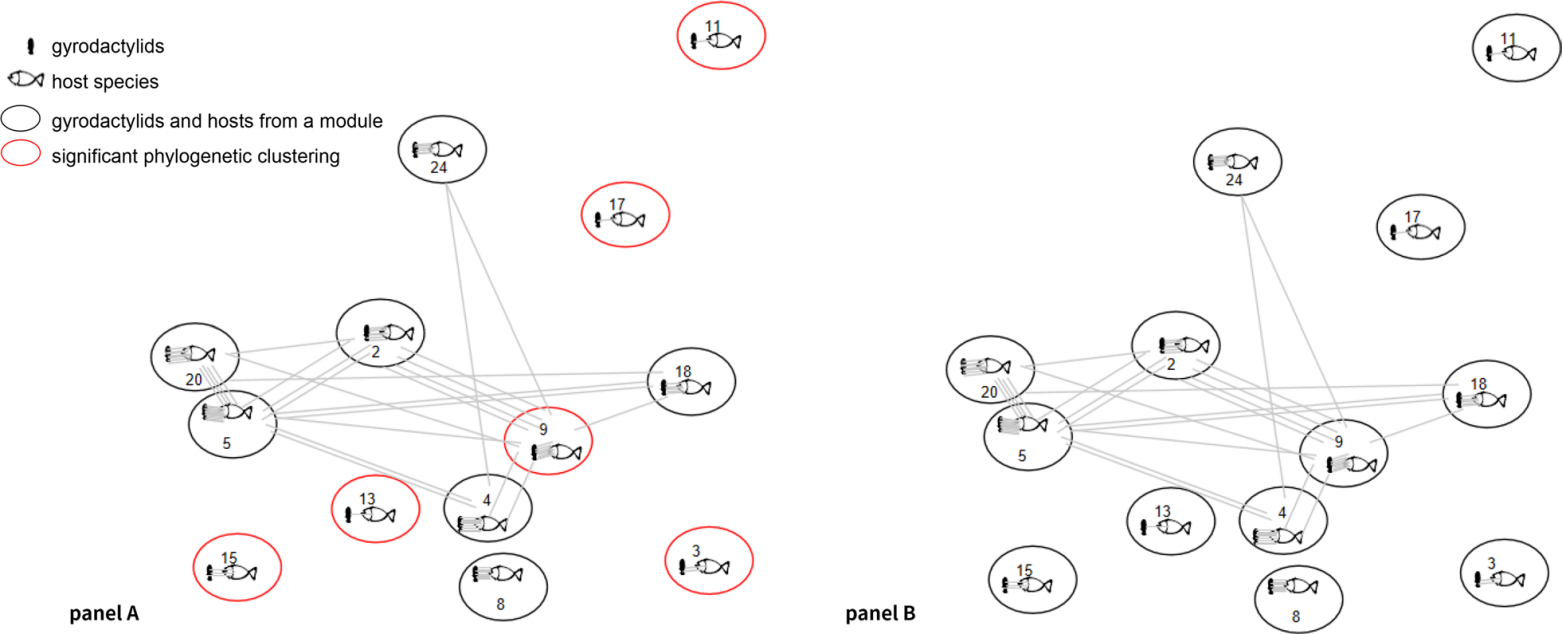
Modular simplification of the interaction network showing statistical significance in the phylogenetic module structure. A network module can be defined as a group of nodes that shares more links within them than with nodes from other modules. In the figure, a module is represented by an ellipse containing icons of a gyrodactylid and a fish. Panel A: six modules that were formed by phylogenetically clustered gyrodactylids have red circles, and others have black circles. Panel B: after accounting for geography, none of the modules exhibited a significant phylogenetic clustering. The module number is displayed in the circle. Gray lines represent the edges of the interaction network

The interaction network identifies groups (modules) of hosts and gyrodactylids that tend to interact more among themselves than with others [57]. The interaction network describing the host use by gyrodactylids was significantly modular (Q = 365.633, *p* < 0.010). It was divided into 41 modules, of which seven were formed by more than four gyrodactylids (Additional file 3: Table S3). In total, these seven modules represented 50.5% of the gyrodactylids analyzed. The remaining modules were mostly composed of only one gyrodactylid species. Using an index of phylogenetic distinctiveness, we found that gyrodactylids were significantly phylogenetically clustered in six out of 41 modules (Fig. 2). Only one of those (Module 9) comprised more than four gyrodactylids.

Using the same network approach as for the host use, we characterized the geographical occurrence of gyrodactylid species. The structure of this geographical network was also significantly modular (Q = 145, *p* < 0.010), comprising five modules (Additional file 3: Table S4).

Gyrodactylids from the same host use-based interaction modules were not more closely phylogenetically related than gyrodactylids across different modules occurring in the same geographical area. In other words, the geographic distribution of gyrodactylids exhibited a significant contribution to the phylogenetic structure of interaction modules (Fig. 2 and Additional file 3: Table S5).

To determine the role of phylogeny and geography in the host use of gyrodactylids, we evaluated the impact of geography and phylogeny in effectively shaping the differences in host use across gyrodactylid species using a generalized regression method. The coefficients of both independent variables and their interactions were highly significant in the built-in t-test (Table 4). However, when we compared 500 random interaction matrices to evaluate the significance of the independent variable coefficients, geography was the only significant factor affecting the host use patterns (Table 4).

**Table 4:** Influence of phylogeny and geography on shaping the host use in gyrodactylids

Factor	Coefficient	Pr ( >| t |)
Geography	0.215066*	< 0.001
Phylogeny	0.075465	< 0.001
Shared	− 0.075025	< 0.001

The magnitude of the coefficient represents the degree of influence of the independent variable on the dependent variable. “Shared” represents the interaction between geography and phylogeny. The 'Pr ( >| t |)' column shows the significance of the coefficient inferred using the glm function in R. The * symbol indicates that the coefficient was larger than 95% of the null cases from 500 random interaction matrices

## Discussion

Co-speciation events are relatively rare and difficult to identify with confidence, i.e. susceptible to methodological artifacts [21]. Previous studies of coevolution between gyrodactylids and hosts focused on only a few specific groups of fish hosts and their gyrodactylid parasites [21, 22] and molecular markers with rather low resolution. To improve the resolution of our analyses, we employed a much wider scope of species and for the first time attempted to employ a multilocus dataset (mitogenome). Furthermore, to make sure that our findings were not methodological artifacts, we compared the results of four different software programs, two of which were topology based and two distance based. The results consistently suggest the existence of a highly significant coevolutionary relationship (topological congruence) between gyrodactylids and their hosts, regardless of the algorithm and dataset used.

We used two different types of data, each of which has unique advantages and disadvantages. Whereas the *18S rRNA* offers far better species coverage, the unstable topology and low support values in the *18S* + *ITS* phylogenies of gyrodactylids indicate that these sequences contained too weak a phylogenetic signal to completely resolve the phylogeny. Compared to this dataset, mitochondrial genomes carry a much larger amount of phylogenetic signal, which resulted in more stable topologies with higher support values. However, they offered much lower species coverage.

### Coevolutionary events of gyrodactylids and their fish hosts

Events such as population isolation, speciation, extinction, and host switch can all affect the coevolution of host and parasite populations [63]. Our PHMITOS dataset analyses suggest that duplication & host switch was the most common coevolutionary event in gyrodactylids. However, in the P18SHMITO dataset, loss and failure to diverge were the most common coevolutionary events, whereas duplication & host switch was merely the third most common event. Besides, results showed that the combined number of duplications and duplication & host switch events was much higher than the number of cospeciation events in both datasets, which was particularly pronounced in the P18SHMITO dataset. The occurrence of host switch events is closely associated with the ability of gyrodactylids to transmit between hosts continuously throughout their lifetime. It has been proposed that opportunities for host switch would be increased as the opportunity for contact with new host species is increased for gyrodactylids, in contrast to species in which transmission occurs only at the larval stage [18]. We furthermore divided the host switch-related events into the S-type host switch (host switch followed by speciation) and the NS-type host switch (host switch not followed by speciation) (for more details, see Additional file 2). Both types are related to the time scale of differentiation and depend on whether there is gene flow between gyrodactylids after the host switch.

In addition, the loss and failure to diverge events could also have a large impact on the reconstruction of host-parasite coevolutionary history. A “failure to diverge” event occurs when a host speciates and the parasite maintains the ability to infect both host species. All Jane 4 results indicated that “failure to diverge” events were relatively common in gyrodactylids, although less common than loss events. A large number of failure to diverge events could be explained by the NS-type host switch, which indicates the existence of a continuous gene flow between parasite populations infecting different hosts, thus preventing speciation of gyrodactylid parasites into two different species. We hypothesize that this might be related to the peculiar contact transmission of gyrodactylids (for more detail, see Additional file 2). This may explain why co-speciation events are not dominant in our results.

The loss events refer to situations wherein host species succeeded in permanently evading a gyrodactylid parasite. Such events may occur when the ancestral host fish species undergo speciation into two species, but the gyrodactylid parasite associated with the ancestral host species is only retained in one of the two descendant host lineages. A hypothetical scenario might involve the differentiation of fish hosts due to geographic isolation (allopatric speciation), wherein the host manages to escape its parasite, the parasite being unable to adapt to the new environment, or accidentally because of the host undergoing a strong population bottleneck, for example, if none of the founding members of the new host population are infected by the gyrodactylid parasite, or if the parasite fails to spread through the new population because of very low population density, and eventually becomes locally extinct. These escapes may benefit the fish hosts by reducing their parasite load. In a scenario where the two host populations come into contact again after having undergone speciation, if gyrodactylids fail to infect the naive population because of high levels of adaption to the paternal host population, the large number of loss events identified in our study may be interpreted as further evidence for the high host specificity of gyrodactylids. Finally, it should be stressed that some of the loss events identified in this study are likely to be artifacts caused by incomplete sampling and identification of fish parasites in the wild [64].

### Host switch

Although we found strong evidence that gyrodactylids parasitizing more than one host are much more likely to parasitize closely related host species than distantly related ones, our results also indicate that a small proportion of gyrodactylids may actually be able to infect distantly related hosts. Indeed, some of these outliers have been recognized before. For example, *Gyrodactylus salaris* can infect fish species belonging to two different orders, Salmoniformes and Cypriniformes [65]. *Gyrodactylus arcuatus* can infect both the three-spined stickleback (order Gasterosteiformes) [66] and gobies (order Gobiiformes) [67], whereas *Gyrodactylus flesi* parasitizes on fishes from both Chondrichthyes and Actinopteri classes [25]. However, the prerequisite for this is either that these phylogenetically distant hosts co-exist geographically or that artificial introductions occur. As expected, we found that these phylogenetically distant fishes exhibited overlapping geographical distributions, which facilitated these major host switches (Additional file 3: Table S6).

**Fig. 3:**
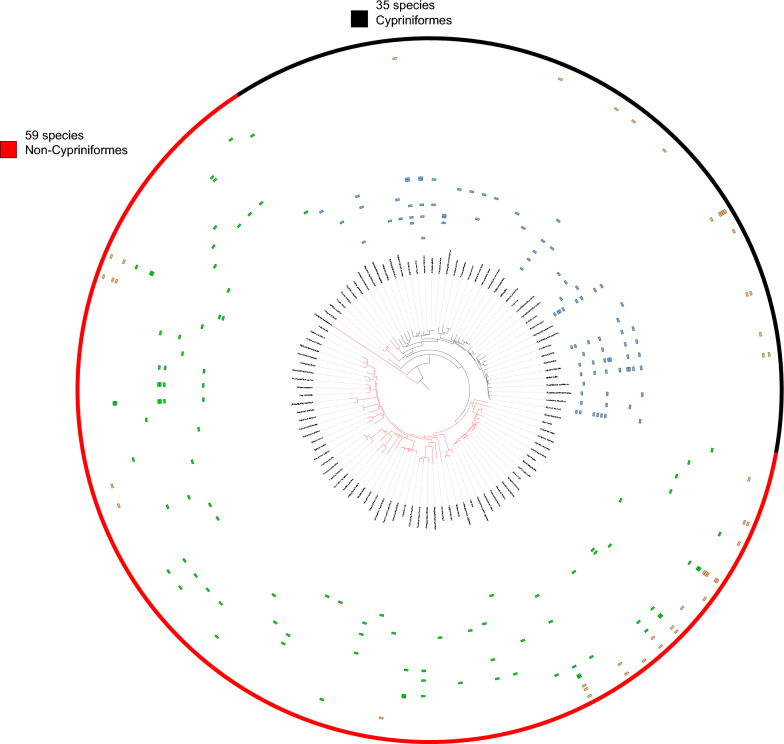
The phylogeny of hosts (fish) depicting the associations with gyrodactylid parasites. Non-Cypriniformes hosts are indicated by the red semi-ring, and Cypriniformes hosts are indicated by the black semi-ring. Blue: gyrodactylids parasitising only Cypriniformes hosts. Brown: gyrodactylids parasitising both Cypriniformes and non-Cypriniformes hosts. Green: gyrodactylids parasitising only non-Cypriniformes hosts. Numbers above the trees depict the total number of gyrodactylid species parasitizing on Cypriniformes (values to the right) and non-Cypriniformes (values to the left) hosts

Malmberg [68] conducted an early research on the origin of Gyrodactylidae and proposed that primitive gyrodactylids parasitize primitive fish species, while the most advanced gyrodactylids parasitize the most advanced fish species. According to the phylogenetic relationships available at that time, Boeger et al. [18] found that Gyrodactylidae originated in the freshwater environments of South America as parasites of armored catfishes (Siluriformes: Loricariidae), but the oldest species within the *Gyrodactylus* genus (such as *Gyrodactylus elegans*) were all parasitic on Cypriniformes fish hosts. Based on the existing biogeographical and phylogenetic reconstruction evidence, we propose that *Gyrodactylus* probably originated on Cypriniformes hosts. First, the genus *Gyrodactylus* appeared relatively early in the evolutionary history of the family Gyrodactylidae, approximately 210 million years ago (albeit with wide confidence intervals, and results varied among datasets) [18, 68, 69], which approximately coincides with the origin of Cypriniformes approximately 193 million years ago (also with wide confidence intervals) [70]. Furthermore, a rapid increase in species diversity of the Cypriniformes occurred approximately 100 million years ago [70], which also coincides with a similar pattern observed in the genus *Gyrodactylus* [69]. In addition, Cypriniformes were also hosts of basal lineages within the *Gyrodactylus* clade (such as *Gyrodactylus elegans* and *G. prostae*) in our dataset. The Jane 4 algorithm identified several major host switches (Cypriniformes to non-Cypriniformes) taking place at the root of Cypriniformes (Additional file 1: Figure S15). Assuming the origin of *Gyrodactylus* on Cypriniformes hosts, on this basis we propose four major host switch events from the ancestral Cypriniformes hosts to non-Cypriniformes hosts, mostly to Salmoniformes. This timing may help explain the mechanism behind these host switch events, as it is possible that they occurred deep in the evolutionary history, while these host lineages still shared a relatively recent common ancestor and thus exhibited a much higher level of similarity than today. Notably, this is the first time that multiple cross-order host switch events were inferred in gyrodactylids. Previously, Zietara et al. [71] identified host switches at the family level and hypothesized that these switches triggered the adaptive radiation of several *Gyrodactylus* lineages. As these major (interordinal) host switch events are relatively rare, the existence of numerous gyrodactylid lineages that parasitize non-Cypriniformes hosts can be attributed to adaptive radiation following major host switch events. This is evidenced by the fact that 57.28% of gyrodactylid species included in our dataset parasitize only non-cyprinid hosts, i.e. a majority of species can be directly traced to these few major host switch events (Fig. 3). Our results suggest that host switch plays a more important role in the coevolution process of gyrodactylids and their fish hosts than co-speciation and provides strong support to the previous finding that an adaptive mode of speciation associated with host switch is more prevalent than co-speciation among the gyrodactylids [18]. This finding also supports the proposal of a recent model study, which suggested that host switch can favor speciation in parasites [72].

### Host specificity

Phylogenetically closely related host species are more likely to have similar ecological, physiological, and immune properties, which makes them more likely to be colonized by the same or closely related parasites. Concordantly, closely related parasites tend to have similar hosts and host specificity. Many authors emphasized the importance of host specificity in host and parasite systems and argued that only obligate parasites with strong host specificity can reflect the internal mechanism of host-parasite coevolution [10, 73]. While several previous studies used the number of host species infected by the parasitic species as the working definition of host specificity, herein we also accounted for phylogeny and geography to calculate host specificity. To test the hypothesis that host specificity and coevolution levels are positively correlated, we conducted a correlation analysis between the phylogenetic host specificity and *p* values of individual links inferred using the ParaFit statistics. We rejected the hypothesis, as these two parameters were not correlated. Finally, nonparametric tests indicated that gyrodactylids with relatively high host specificity did contribute more to the overall coevolutionary pattern than those with low host specificity.

**Fig. 4:**
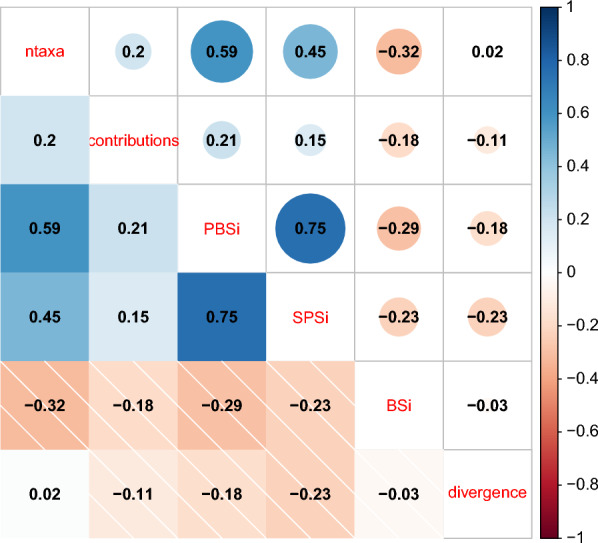
Visualization of the correlation matrix of four kinds of host specificity, divergence time, and *p* values of host-parasite individual links. Blue: a positive correlation; red: a negative correlation. SPS_i_: phylogenetic host specificity; PBS_i_: phylobeta host specificity; ntaxa: basic host specificity; contributions: *p* values of host-parasite individual links; BS_i_: geographic host specificity; divergence: divergence time of gyrodactylids

The correlations between the basic, phylogenetic, and phylobeta host specificity were relatively high, whereas geographic host specificity exhibited a low correlation with these three types of host specificity (Fig. 4). Regarding the SPS_i_, positive values indicate low phylogenetic specificity (i.e. greater host phylogenetic diversity than expected by chance), whereas negative values indicate high phylogenetic specificity [11]. According to this criterion, our results showed that gyrodactylids had relatively high host specificity and intermediate geographic host specificity. Moreover, the vast majority (94.2%) of gyrodactylids exhibited SPS_i_ values < 0, which indicates that gyrodactylids tend to parasitize on phylogenetically related hosts. However, some species exhibited low host specificities, which complicated the comparisons of the host and parasite phylogenies in TreeMap 3 and Jane 4 analyses. To address this problem, we eliminated the top 15% of gyrodactylids with the highest SPS_i_ values and re-conducted analyses on this subset of data. Results indicate that removing the gyrodactylids with high SPS_i_ values had a smaller impact on the coevolutionary history reconstruction than removing the 17 hosts parasitized by more than three gyrodactylids. Specifically, two types of events, loss and failure to diverge, both sharply decreased, as did the global cost of the coevolutionary scenario (Table 3).

We finally conducted a correlation analysis of the divergence time of gyrodactylids and the SPS_i_ value and found that evolutionary younger gyrodactylid parasites exhibit lower phylogenetic host specificity. This result is inconsistent with the hypothesis that there is a general evolutionary trend towards generalism in parasites, which proposes that the earlier the parasite differentiated, the more likely it is that the parasite evolved into a generalist [74, 75].

The key to understanding the evolution of host-parasite associations is that selection for the increased specialization of parasites to their hosts constrains host use and promotes speciation. However, switches onto relatively unrelated hosts are a common phenomenon in phylogenetic diversification of parasite lineages [76]. The niche theory predicts that specialists will be more sensitive to environmental perturbation compared to generalists, a hypothesis well supported in free-living species, and there is also some support for this in parasites [77]. However, McCoy et al. [78] speculated that due to the close association between parasites and their hosts, many apparently generalist parasites have a high potential to become specialized for different host species, i.e. host-related selection pressures may cause specialization in previously generalist parasites. They found evidence that ectoparasites become more specialized after a host switch. In light of the evidence from this and previous studies that host switch is an important speciation mode that affects the coevolution of fish hosts and their gyrodactylid ectoparasites [18], the weak negative correlation coefficient inferred in our analyses may be a consequence of host switches and corresponding host-related selection pressures. However, further research is needed to prove this hypothesis.

### Geographic distribution

**Fig. 5:**
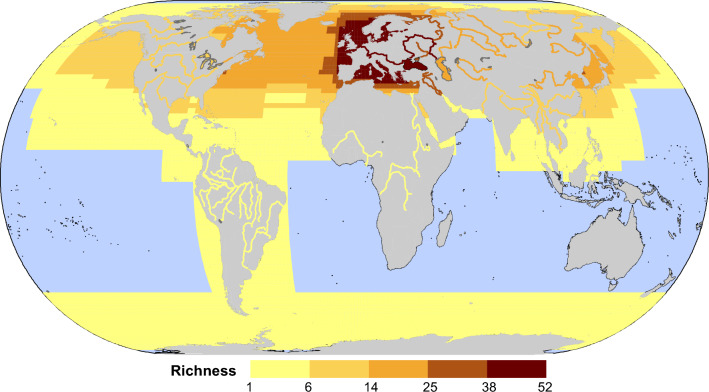
Geographical distribution of host fishes. Richness: the number of fish species in the distribution area

Gyrodactylids have been reported from 19 orders of bony fishes, which is the widest host range among the monogenean genera [14]. Geographic distribution statistics of fish hosts of gyrodactylids revealed two main clusters: the Mediterranean and Western Europe (Fig. 5). Such cluster distribution of hosts may create favorable conditions for the contact transmission and host switch of gyrodactylids, the prerequisite for which is that the hosts have overlapping geographical distributions. This may explain our finding that the number of duplication & host switch and failure to diverge events is much higher than the number of co-speciation events (Jane 4 results). This is also consistent with a previous finding that simultaneous co-speciation of parasite and host is comparatively rare among the gyrodactylids, but that host switch is common [14]. The geographic distribution of fish hosts is also a suitable explanatory variable for major host switch events. For example, gyrodactylids such as *Gyrodactylus phoxini* and *G. salaris* parasitize on both Cypriniformes and non-Cypriniforme fishes, but their hosts are distributed in the same area. Besides, we found that host fishes of gyrodactylids had overlapping geographical distributions in cases where we identified failure to diverge events. For example, *Gyrodactylus carassii* and *G. laevis* both parasitize on multiple sympatric hosts (e.g. *Leucaspius delineatus* and *Alburnus alburnus*), which suggests that the inferred failure to diverge events of these two parasitic species can be attributed to overlapping distributions of these two hosts in Europe.

### Network analysis: phylogeny vs. geography

**Fig. 6:**
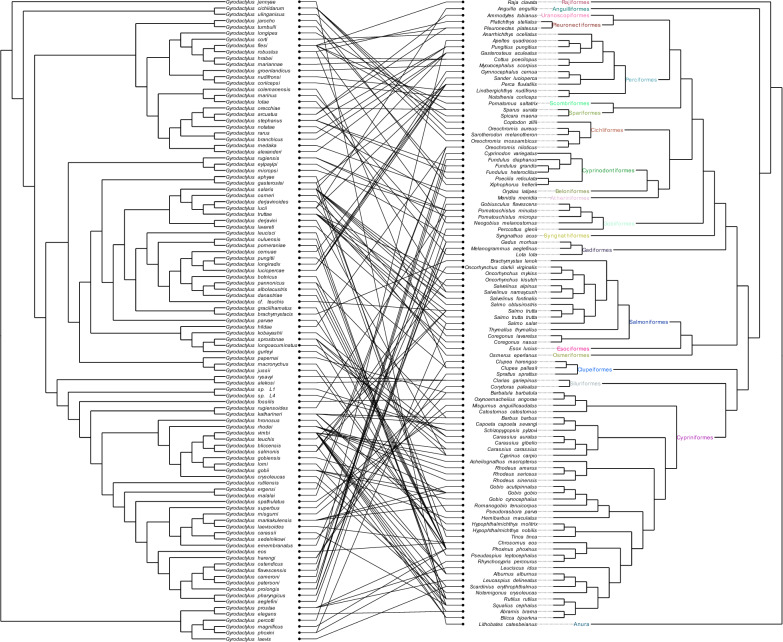
The tanglegrams of gyrodactylids and host fishes inferred using the P18SHMITO-ML dataset. The figure contains a parasite tree (on the left), a host tree (on the right) with the order-level annotation, and a set of host-parasite associations (the host range of each parasite)

As mentioned earlier, gyrodactylids infect a broad range of hosts and encompass both highly host-specific and generalist species (Figs. 1 and 6). We also found that most hosts of the latter group co-exist geographically. Following this, using multiple analyses, we highlighted the importance of biogeographical processes as determinants of patterns of host use in gyrodactylids, which had not been systematically studied before. Traditionally, phylogenetic constraints on patterns of host use by parasites received far more scientific attention than the role of geographic distribution [62]. Since related species tend to inhabit the same regions, they mostly interact with species that also co-occur in those regions. This may produce a pattern of phylogenetically conserved interactions regardless of the actual existence of evolutionary constraints on host use. Further combining our network analysis results with the fact that colonization of gyrodactylids to novel hosts can happen in the same region despite hosts being phylogenetically distant (e.g. we identified several major host switch events in our study), it appears that gyrodactylid adaptation to novel host lineages is more restricted by geographical than by evolutionary processes. This also suggests that gyrodactylids are evolutionarily relatively flexible and able to colonize distantly related new hosts as long as they are sympatric and a sufficiently wide window of evolutionary time is provided.

## Conclusions

Based on multiple co-phylogenetic and co-speciation analyses, our results indicate that the coevolutionary relationship between gyrodactylids and their hosts was highly significant no matter which software algorithm or topology was used. The analyses indicate that gyrodactylids with high host specificity contributed more to the overall pattern of coevolution than those with low host specificity. Our analyses support the view that speciation by host switch is an important speciation mode and that host switch events affected the coevolution of gyrodactylids and their fish hosts. We also found that major host switches are relatively rare events that are greatly influenced by geographical factors, but they may produce opportunities for major radiation events, as long as the colonization is successful. We also discussed the potential roles of biogeographical factors in various types of coevolutionary events. In our dataset, evolutionary patterns in host use by gyrodactylids were largely determined by the geographical distribution of hosts and parasites, but with some limitations. Finally, we found that both gyrodactylids with low host specificity and hosts infected by multiple gyrodactylid species (the latter particularly strongly) may interfere with the host-parasite coevolutionary reconstruction. In a sentence, our findings suggest that the coevolution of *Gyrodactylus* flatworms and their hosts is largely driven by geography, phylogeny, and host switches.

### Supplementary Information

**Additional file 1: Figure S1:** The complete mitochondrial genomes of *Gyrodactylus *sp. L1 and *Gyrodactylus *sp. L4. **Figure S2: **Gyrodactylid phylograms inferred using the PHMITOS dataset. Panel A: phylogenetic tree constructed using the maximum likelihood method; panel B: phylogenetic tree constructed using the Bayesian inference method. **Figure S3: **Host phylograms inferred using the PHMITOS dataset. Panel A: phylogenetic tree constructed using the maximum likelihood method; panel B: phylogenetic tree constructed using Bayesian inference method. **Figure S4:** Gyrodactylid phylogram inferred using the ML analysis and P18SHMITO dataset. *Paragyractylus variegatus* was used as the outgroup. **Figure S5:** Gyrodactylid phylogram inferred using the ML analysis and P18SHMITO dataset. The clade comprising *Gyrodactylus laevis*, *G. pecotti*, *G. magnificus*, *G. phoxini*, *G. elegans*, and *G. prostae* was used as the outgroup. **Figure S6: **Gyrodactylid phylogram inferred using the BI analysis and P18SHMITO dataset. **Figure S7: **Host phylogram inferred using the ML analysis and P18SHMITO dataset. From left to right: a tree with bootstrap values = 100% shown as pentagram symbols at nodes, species names, and taxonomy (family, order, and class, respectively). **Figure S8: **host phylogram inferred using the BI analysis and P18SHMITO dataset. From left to right: a tree with bootstrap values = 1.0 shown as pentagram symbols at nodes, species names, and taxonomy (family, order, and class, respectively). **Figure S9: **Treemap 3 tanglegram of the PHMITOS dataset (ML topology). Red dots indicate nodes that exhibited significant congruence between host and parasite topologies. The intensity of the color of the dot is positively correlated to the significance (*p* value) of congruence. **Figure S10: **Treemap 3 tanglegram of the PHMITOS dataset (BI topology). Red dots indicate nodes that exhibited significant congruence between host and parasite topologies. The intensity of the color of the dot is positively correlated to the significance (*p* value) of congruence. **Figure S11:** Treemap 3 tanglegram of the P18SHMITO dataset (ML topology). Red dots indicate nodes that exhibited significant congruence between host and parasite topologies. The intensity of the color of the dot is positively correlated to the significance (*p* value) of congruence. **Figure S12:** Treemap 3 tanglegram of the P18SHMITO dataset (BI topology). Red dots indicate nodes that exhibited significant congruence between host and parasite topologies. The intensity of the color of the dot is positively correlated to the significance (*p* value) of congruence. **Figure S13: **Jane 4 tanglegrams of the PHMITOS dataset (ML topology). **Figure S14:** Jane 4 tanglegrams of the PHMITOS dataset (BI topology). **Figure S15: **Jane 4 tanglegrams of the P18SHMITO dataset (ML topology). **Figure S16: **Jane 4 tanglegrams of the P18SHMITO dataset (BI topology). **Figure S17: **Panel A: The probability that a host pair with a gyrodactylid in common has a particular patristic phylogenetic distance; panel B: probability that a gyrodactylid pair with a host in common has a particular patristic phylogenetic distance. **Figure S18: **Linear fit trendline of the basic host specificity (Y) and phylogenetic host specificity (X).

**Additional file 2: Supplementary information.** Supplementary material, methods, results, discussion and limitations of this study.

**Additional file 3: Table S1:** Detailed information for Supplementary figure S17 (panel A). The “hist” column is the patristic distance range of 10 bins, the “count” column is the number of host pairs of 10 bins, the “host_pair” column lists each host pair in detail, the “parasites” column lists the corresponding gyrodactylids species of each host pair. **Table S2:** Detailed information for Supplementary figure S17 (panel B). The “hist” column is the patristic distance range of 10 bins, the “count” column is the number of host pairs of 10 bins, the “parasite_pair” column lists each parasite pair in detail. **Table S3:** Community detection result of interaction network using the function “Gen Louvain” in MATLAB. **Table S4:** Community detection result of the geographic network using the function “Gen Louvain” in MATLAB. **Table S5:** The RPD and the geography-corrected RPD of each module in the interactive network and the corresponding *p* values. **Table S6:** The geographic distribution of fish hosts and parasites.

## Data Availability

The data and materials underlying this article are available in the article and in its online supplementary material or have been published or archived elsewhere. GenBank accession numbers for the two newly sequenced mitochondrial genomes are OR031076 (*Gyrodactylus* sp. L1) and OR031077 (*Gyrodactylus* sp. L4).
