# Correction: Geography, phylogeny and host switch drive the coevolution of parasitic Gyrodactylus flatworms and their hosts

**DOI:** 10.1186/s13071-024-06223-7

**Published:** 2024-03-13

**Authors:** Hong-Peng Lei, Ivan Jakovlić, Shun Zhou, Xiang Liu, Chuan Yan, Xiao Jin, Bo Wang, Wen-Xiang Li, Gui-Tang Wang, Dong Zhang

**Affiliations:** 1 State Key Laboratory of Herbage Improvement and Grassland Agro-Ecosystems, and College of Ecology, Lanzhou University, Lanzhou, 730000 China; 2 Yangtze River Fisheries Research Institute, Chinese Academy of Fishery Sciences, Wuhan, 430223 China; 3 College of Fishery, Guangdong Provincial Key Laboratory of Aquatic Animal Disease Control and Healthy Culture, Guangdong Ocean University, Zhanjiang, China; 4 Shapotou Desert Research and Experimental Station, Northwest Institute of Eco-Environment and Resources, Chinese Academy of Sciences, 320 Donggang West Road, Lanzhou, 730000 People’s Republic of China; 5 Key Laboratory of Aquaculture Disease Control, Ministry of Agriculture, and Key Laboratory of Breeding Biotechnology and Sustainable Aquaculture, Institute of Hydrobiology, Chinese Academy of Sciences, Wuhan, People’s Republic of China

**Correction: Parasites & Vectors (2024) 17:42** 10.1186/s13071-023-06111-6

Following publication of the original article [1], the following two errors came to the authors' attention: affiliation 5 had been written incorrectly and acknowledgement of 'the Open Fund Project of Key Laboratory of Breeding Biotechnology and Sustainable Aquaculture (2023FB07)' had been omitted from the Funding declaration. The correct funding information is: “This work was supported by the National Natural Science Foundation of China (32102840, 32360927); the Key Project of Natural Science Foundation of Tibet (XZ202301ZR0028G); the National Natural Science Foundation of China (31970408); the Open Fund Project of Key Laboratory of Breeding Biotechnology and Sustainable Aquaculture (2023FB07); the Start-up Funds of Introduced Talent in Lanzhou University (561120206)." These errors have since been corrected in the published article. The authors thank you for reading this erratum and apologize for any inconvenience caused.

## References

[1] Lei H-P, Jakovlić I, Zhou S, Liu X, Yan C, Jin X, Wang Bo, Li W-X, Wang G-T, Zhang D (2024). Geography, phylogeny and host switch drive the coevolution of parasitic Gyrodactylus flatworms and their hosts. Parasit Vectors.

