# Supplementary material for: Geography, phylogeny and host switch drive the coevolution of parasitic Gyrodactylus flatworms and their hosts
Source: Parasit Vectors. 2024 Jan 30;17:42. doi: 10.1186/s13071-023-06111-6 (PMC10825989; doi:10.1186/s13071-023-06111-6)
Supplement: Supplementary file 1 — Additional file 1: Figure S1: The complete mitochondrial genomes of Gyrodactylus sp. L1 and Gyrodactylus sp. L4. Figure S2: Gyrodactylid phylograms inferred using the PHMITOS dataset. Panel A: phylogenetic tree constructed using the maximum likelihood method; panel B: phylogenetic tree constructed using the Bayesian inference method. Figure S3: Host phylograms inferred using the PHMITOS dataset. Panel A: phylogenetic tree constructed using the maximum likelihood method; panel B: phylogenetic tree constructed using Bayesian inference method. Figure S4: Gyrodactylid phylogram inferred using the ML analysis and P18SHMITO dataset. Paragyractylus variegatus was used as the outgroup. Figure S5: Gyrodactylid phylogram inferred using the ML analysis and P18SHMITO dataset. The clade comprising Gyrodactylus laevis, G. pecotti, G. magnificus, G. phoxini, G. elegans, and G. prostae was used as the outgroup. Figure S6: Gyrodactylid phylogram inferred using the BI analysis and P18SHMITO dataset. Figure S7: Host phylogram inferred using the ML analysis and P18SHMITO dataset. From left to right: a tree with bootstrap values = 100% shown as pentagram symbols at nodes, species names, and taxonomy (family, order, and class, respectively). Figure S8: host phylogram inferred using the BI analysis and P18SHMITO dataset. From left to right: a tree with bootstrap values = 1.0 shown as pentagram symbols at nodes, species names, and taxonomy (family, order, and class, respectively). Figure S9: Treemap 3 tanglegram of the PHMITOS dataset (ML topology). Red dots indicate nodes that exhibited significant congruence between host and parasite topologies. The intensity of the color of the dot is positively correlated to the significance (p value) of congruence. Figure S10: Treemap 3 tanglegram of the PHMITOS dataset (BI topology). Red dots indicate nodes that exhibited significant congruence between host and parasite topologies. The intensity of the color of the dot is positively correlated to the [file 13071_2023_6111_MOESM1_ESM.docx]

**Additional file figures**

**Geography, phylogeny and host switch drive the coevolution of parasitic *Gyrodactylus* flatworms and their hosts**

Hong-Peng Lei^1^, Ivan Jakovlić^1^, Shun Zhou^2^, Xiang Liu^1^, Chuan Yan^1^, Xiao Jin^3^, Bo Wang^4^, Wen-Xiang Li^5^, Gui-Tang Wang^5^, Dong Zhang^1^*

^1^ State Key Laboratory of Herbage Improvement and Grassland Agro-ecosystems, and College of Ecology, Lanzhou University, 730000, Lanzhou, China.

^2^ Yangtze River Fisheries Research Institute, Chinese Academy of Fishery Sciences, Wuhan, 430223, China

^3^ College of Fishery, Guangdong Ocean University, Guangdong Provincial Key Laboratory of Aquatic Animal Disease Control and Healthy culture, Zhanjiang, China

^4^ Shapotou Desert Research and Experimental Station, Northwest Institute of Eco-Environment and Resources, Chinese Academy of Sciences, 320 Donggang West Road, Lanzhou 730000, People's Republic of China

^5^ Key Laboratory of Aquaculture Disease Control, Ministry of Agriculture, and State Key Laboratory of Freshwater Ecology and Biotechnology, Institute of Hydrobiology, Chinese Academy of Sciences, Wuhan, People’s Republic of China

* Correspondence: Dong Zhang, State Key Laboratory of Herbage Improvement and Grassland Agro-ecosystems, and College of Ecology, Lanzhou University, 730000, Lanzhou, China; Phone: +86-18717167887; Email: [dongzhang0725@gmail.com](mailto:dongzhang0725@gmail.com)


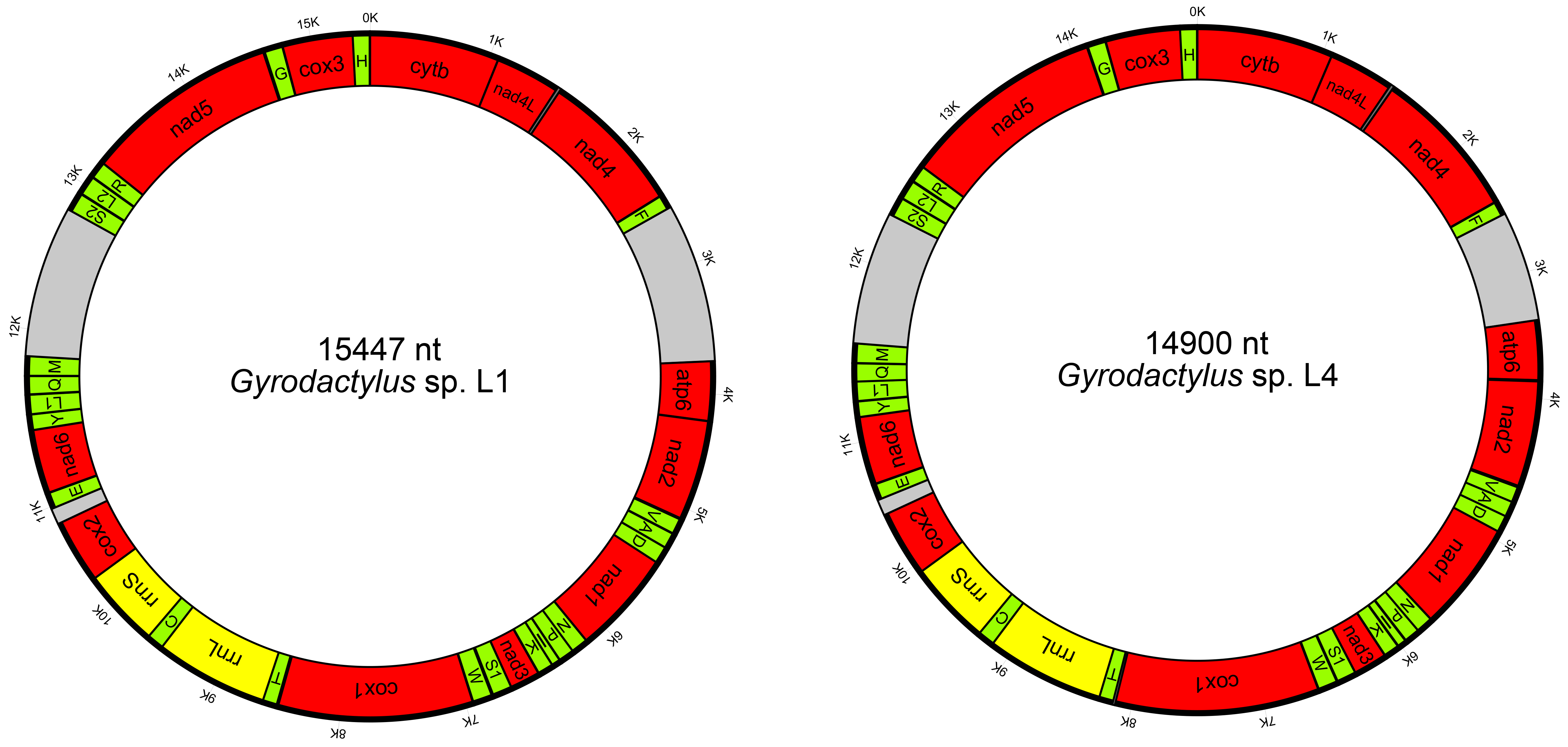


**Additional file 1: Figure S1:** the complete mitochondrial genomes of *Gyrodactylus* sp. L1 and *Gyrodactylus* sp. L4.


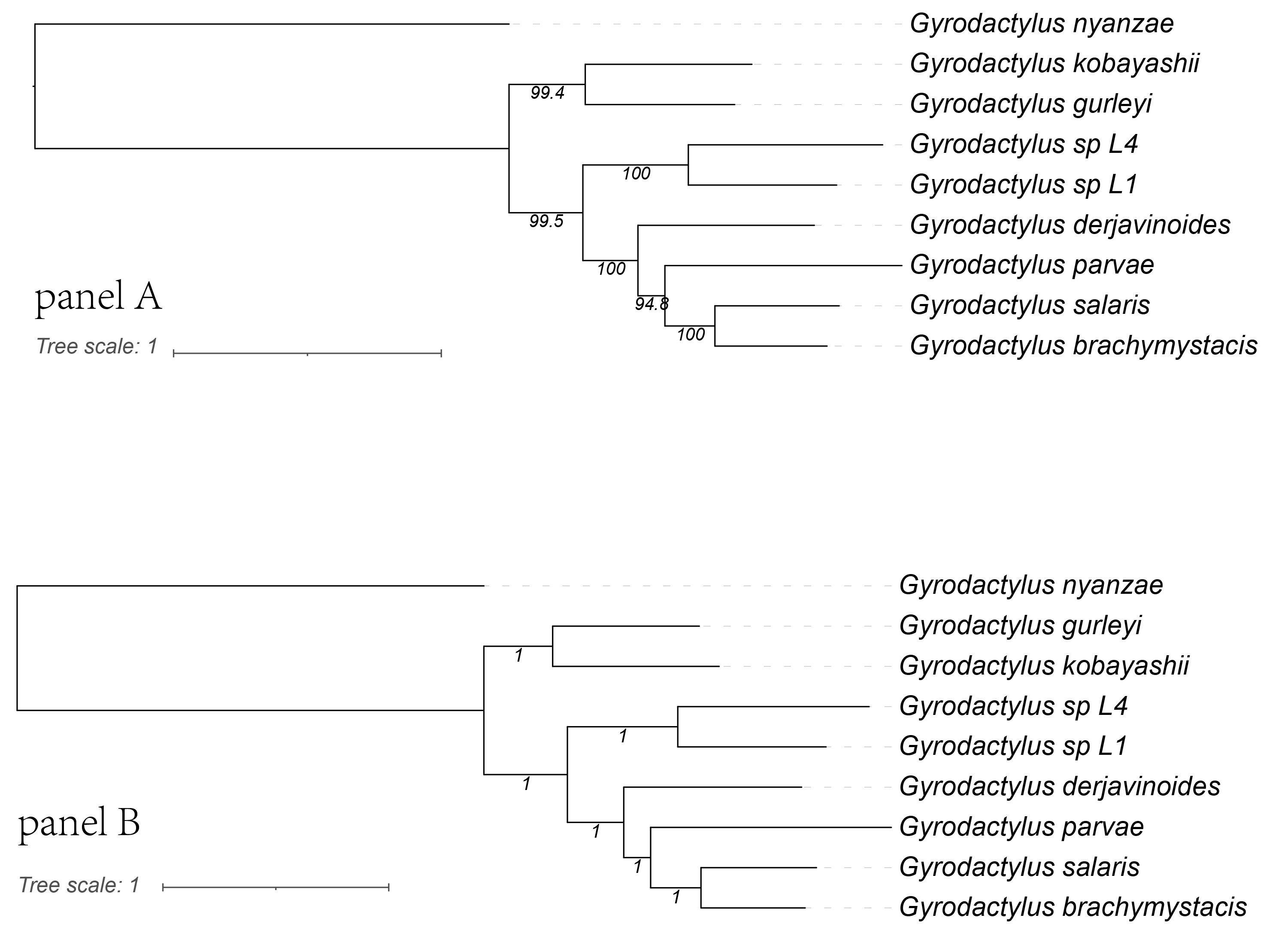


**Additional file 1: Figure S2:** gyrodactylid phylograms inferred using the PHMITOS dataset. panel A: phylogenetic tree constructed using the Maximum-Likelihood method, panel B: phylogenetic tree constructed using the Bayesian Inference method.


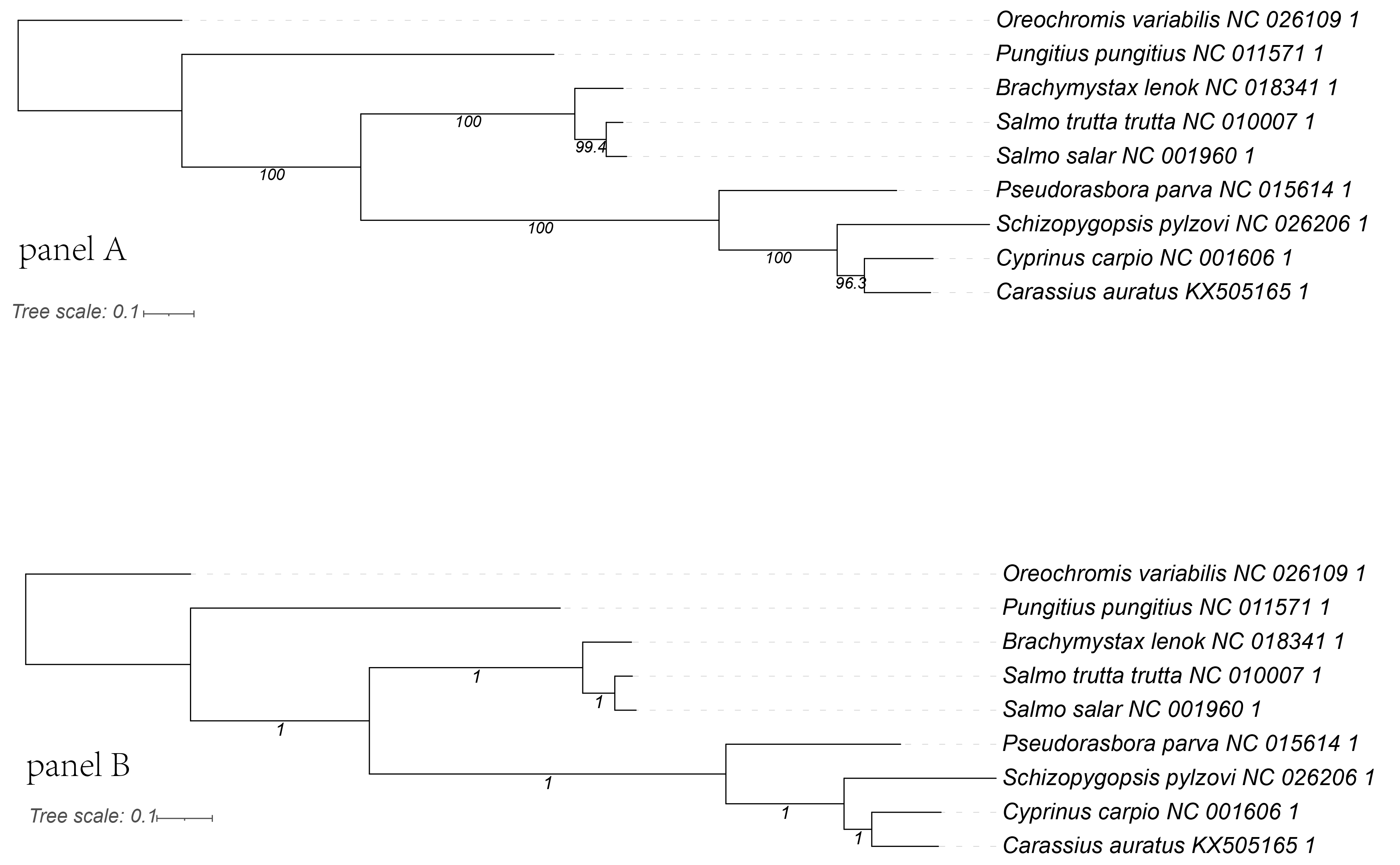


**Additional file 1: Figure S3:** Host phylograms inferred using the PHMITOS dataset. panel A: phylogenetic tree constructed using the Maximum-Likelihood method, panel B: phylogenetic tree constructed using Bayesian Inference method.


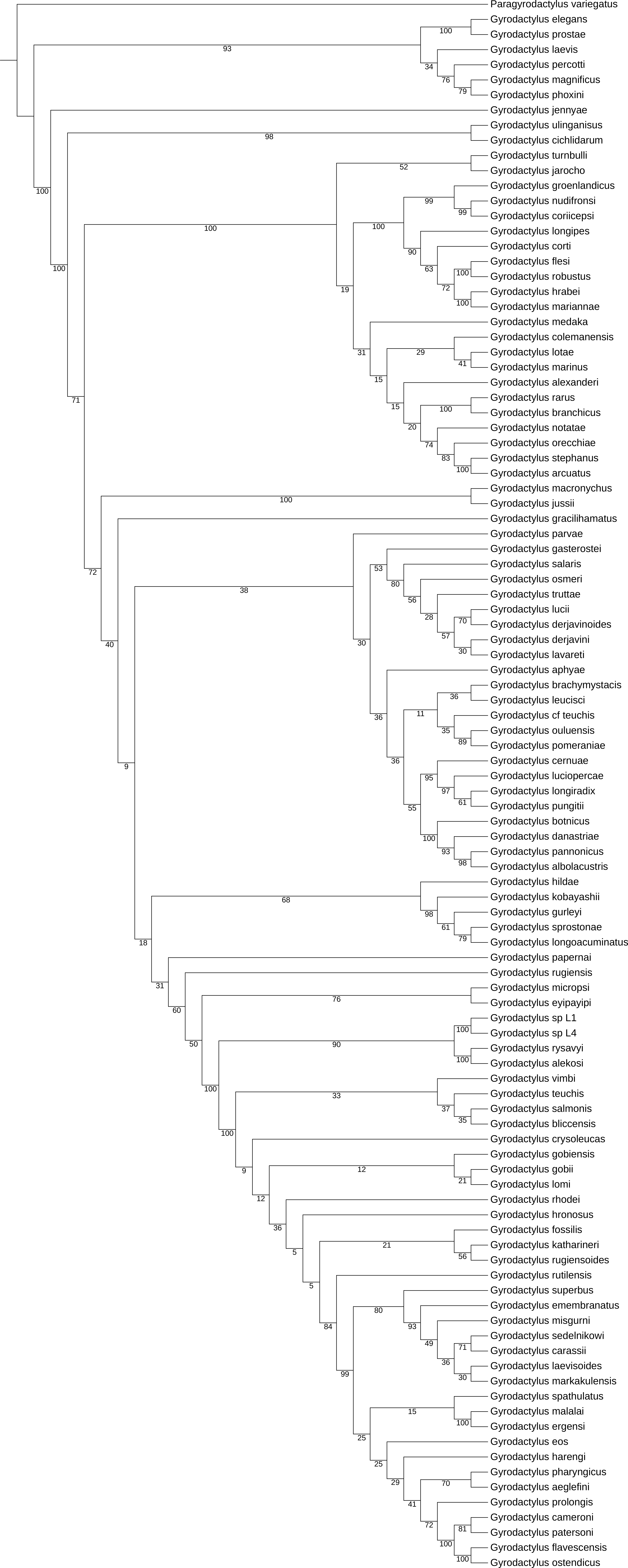


**Additional file 1: Figure S4:** gyrodactylid phylogram inferred using the ML analysis and P18SHMITO dataset. *Paragyractylus variegatus* was used as the outgroup.


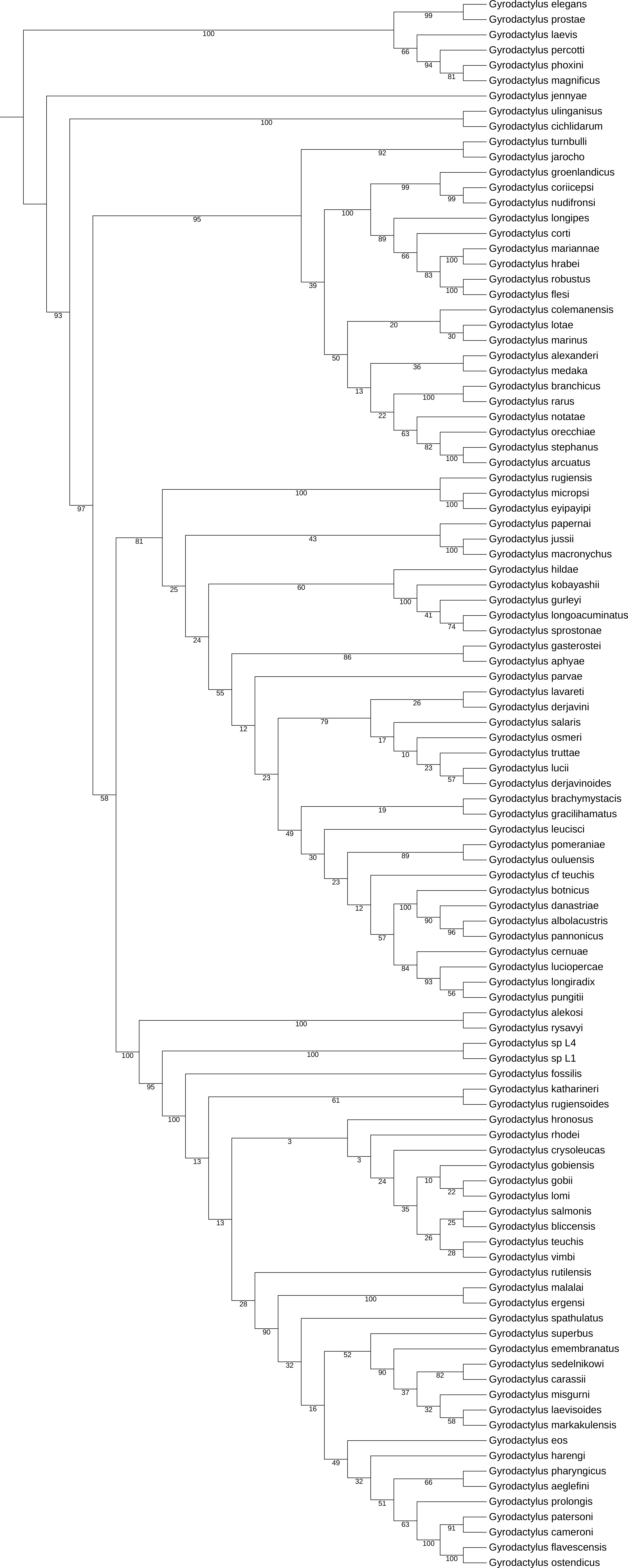


**Additional file 1: Figure S5:** gyrodactylid phylogram inferred using the ML analysis and P18SHMITO dataset. The clade comprising *Gyrodactylus laevis, Gyrodactylus pecotti, Gyrodactylus magnificus, Gyrodactylus phoxini, Gyrodactylus elegans* and *Gyrodactylus prostae* was used as the outgroup.


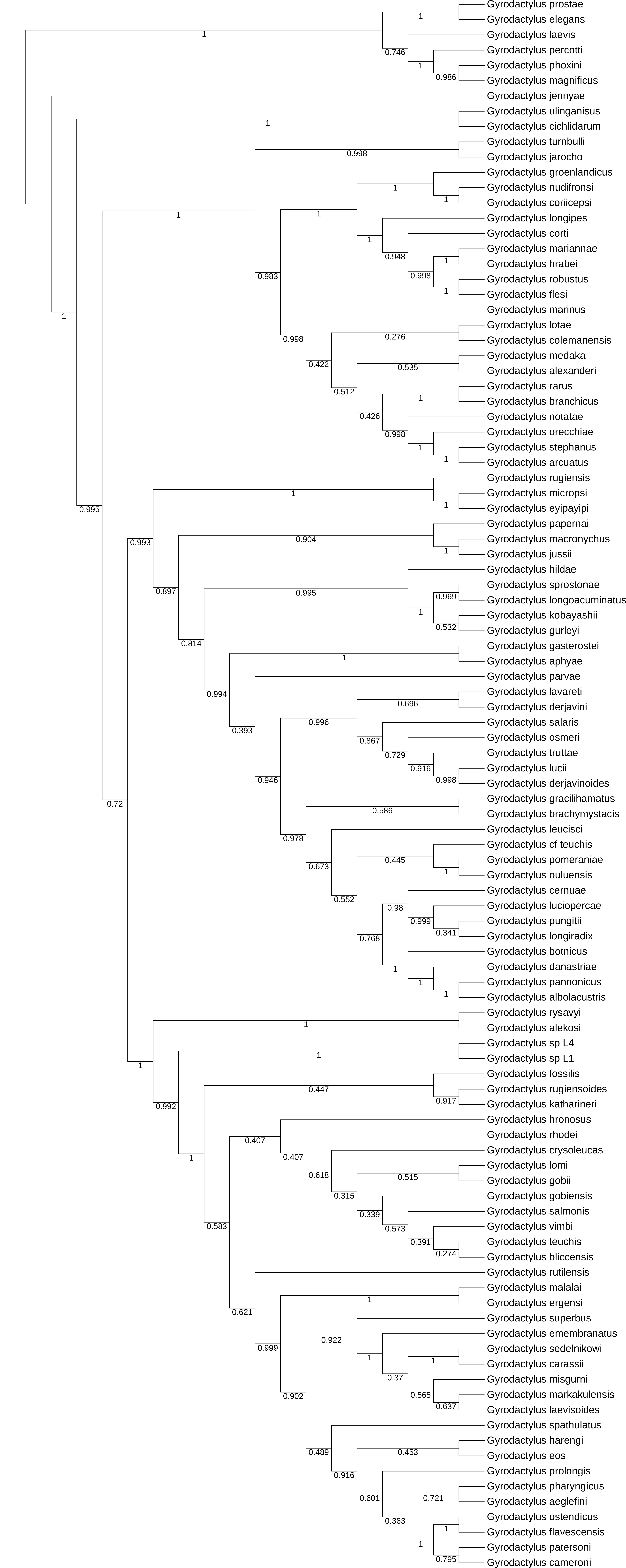


**Additional file 1: Figure S6:** gyrodactylid phylogram inferred using the BI analysis and P18SHMITO dataset.


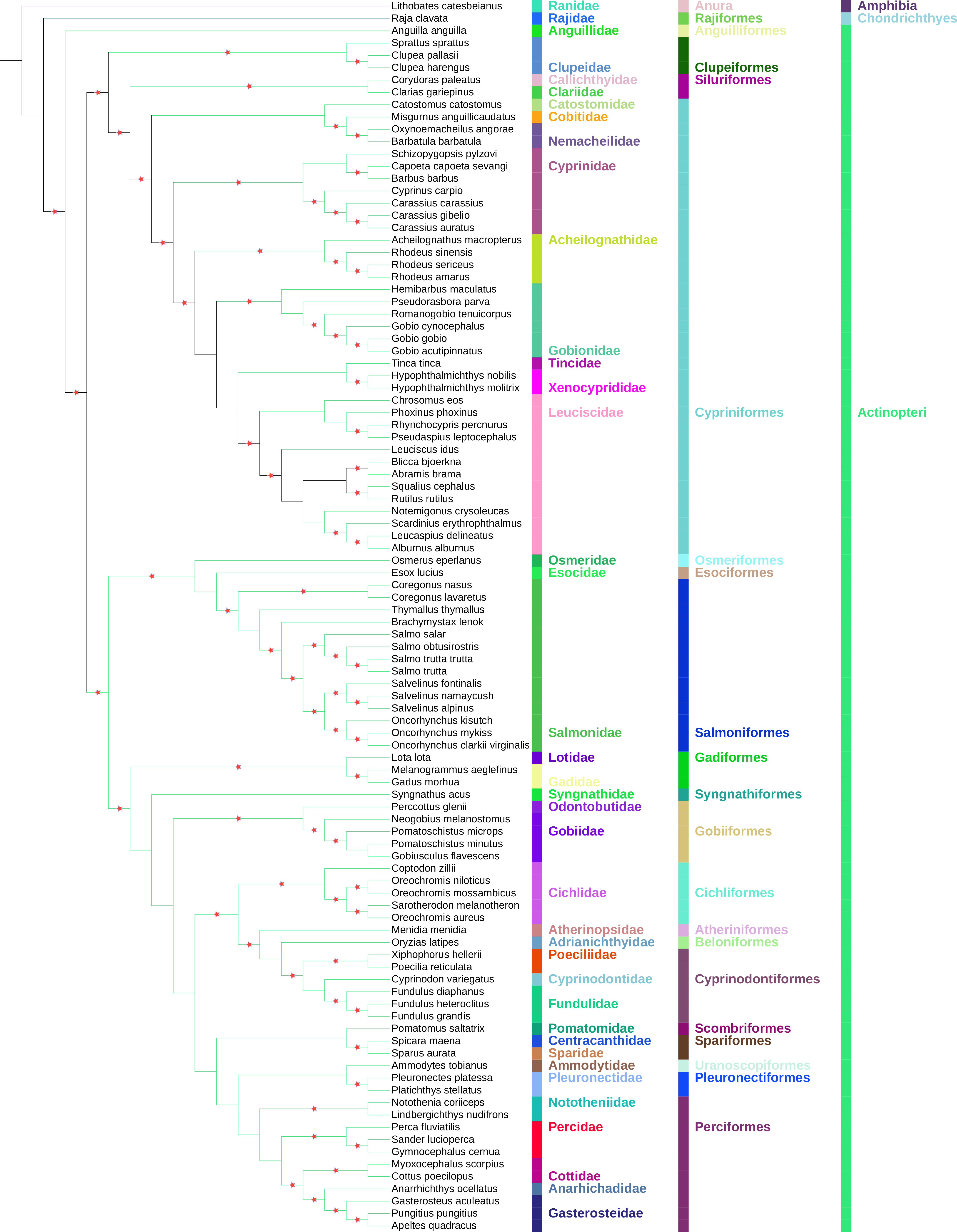


**Additional file 1: Figure S7:** host phylogram inferred using the ML analysis and P18SHMITO dataset. The Figure shows (from left to right): a tree with bootstrap values = 100% shown as pentagram symbols at nodes, species names, and taxonomy (family, order and class, respectively).


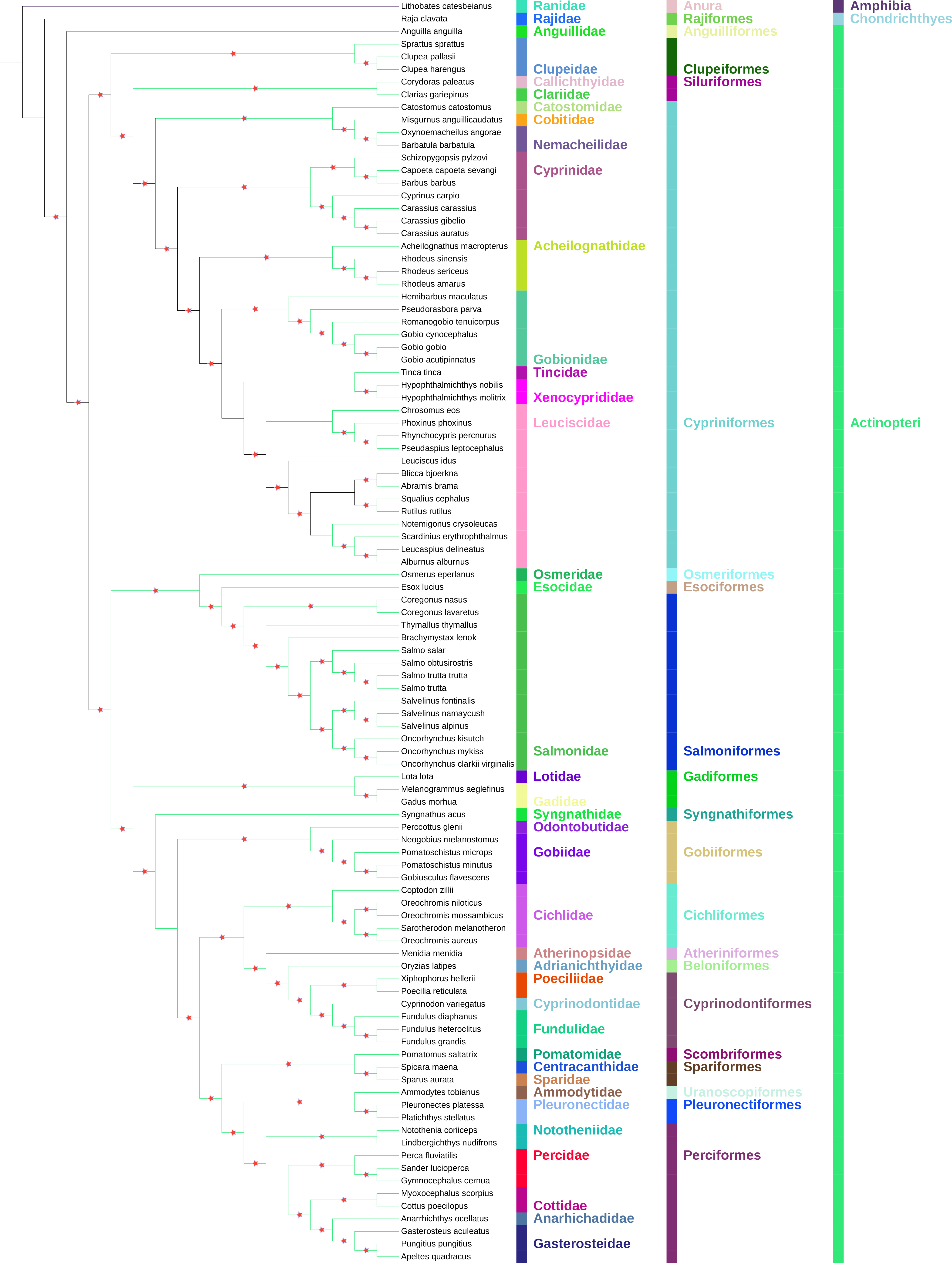


**Additional file 1: Figure S8:** host phylogram inferred using the BI analysis and P18SHMITO dataset. The Figure shows (from left to right): a tree with bootstrap values = 1.0 shown as pentagram symbols at nodes, species names, and taxonomy (family, order and class, respectively).


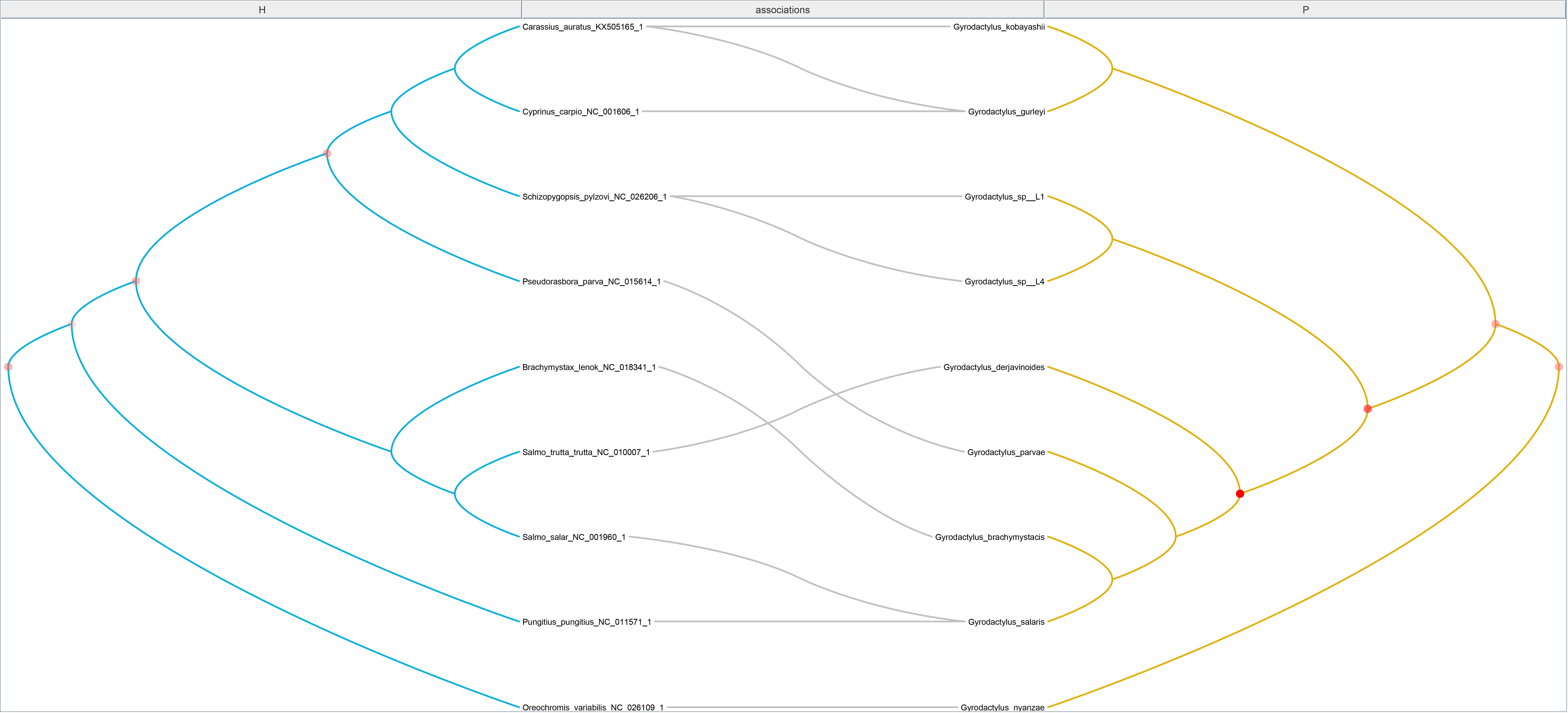


**Additional file 1: Figure S9:** the Treemap 3 tanglegram of the PHMITOS dataset (ML topology). Red dots indicate nodes that exhibited significant congruence between host and parasite topologies. The intensity of the colour of the dot is positively correlated to the significance (p value) of congruence.


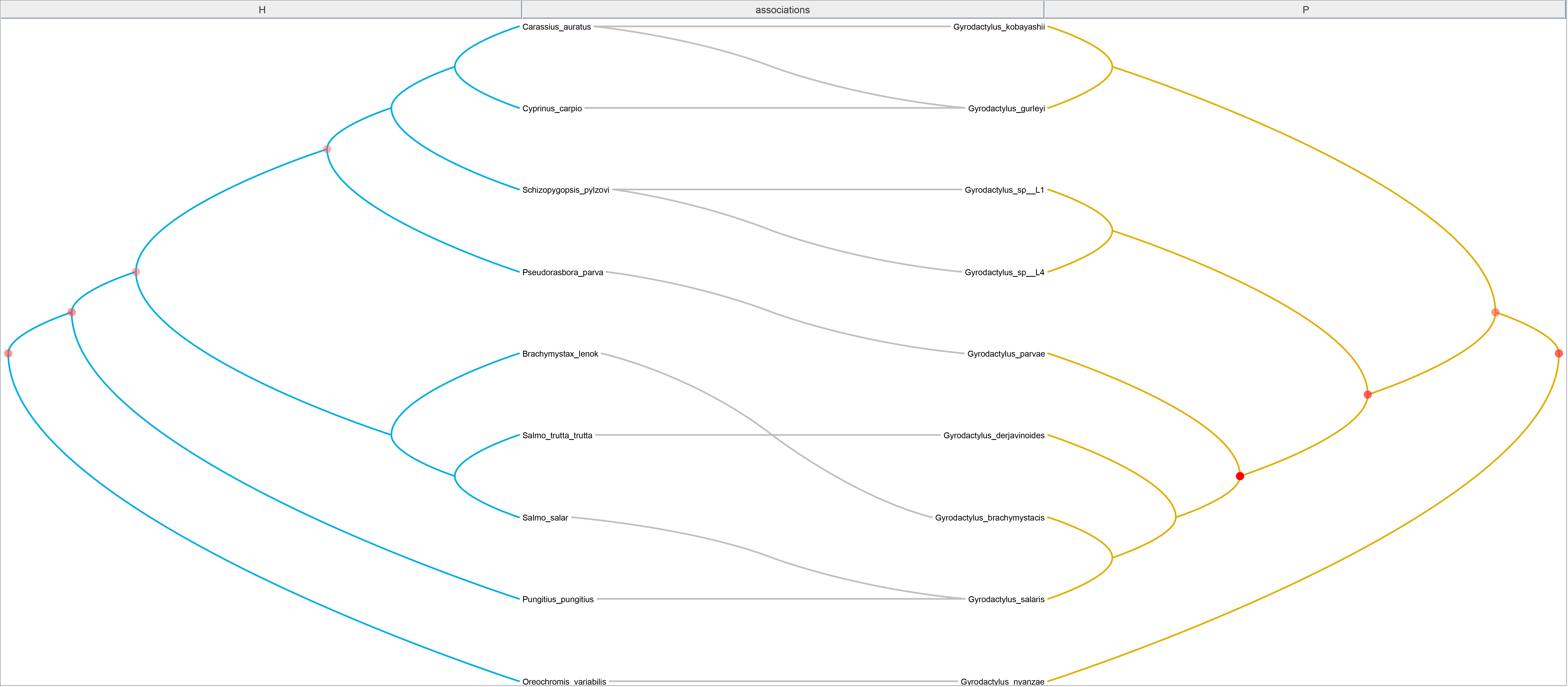


**Additional file 1: Figure S10:** the Treemap 3 tanglegram of the PHMITOS dataset (BI topology). Red dots indicate nodes that exhibited significant congruence between host and parasite topologies. The intensity of the colour of the dot is positively correlated to the significance (p value) of congruence.


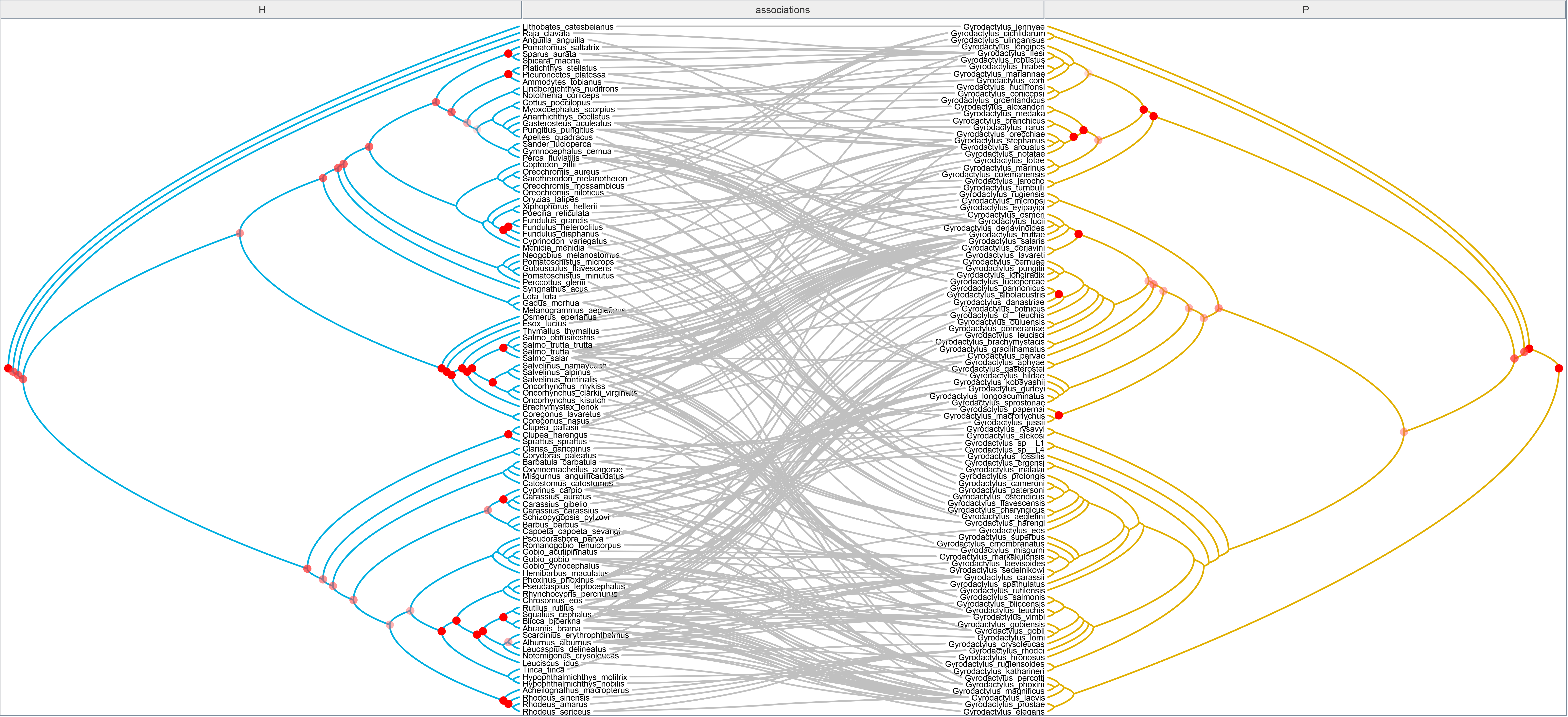


**Additional file 1: Figure S11:** the Treemap 3 tanglegram of the P18SHMITO dataset (ML topology). Red dots indicate nodes that exhibited significant congruence between host and parasite topologies. The intensity of the colour of the dot is positively correlated to the significance (p value) of congruence.


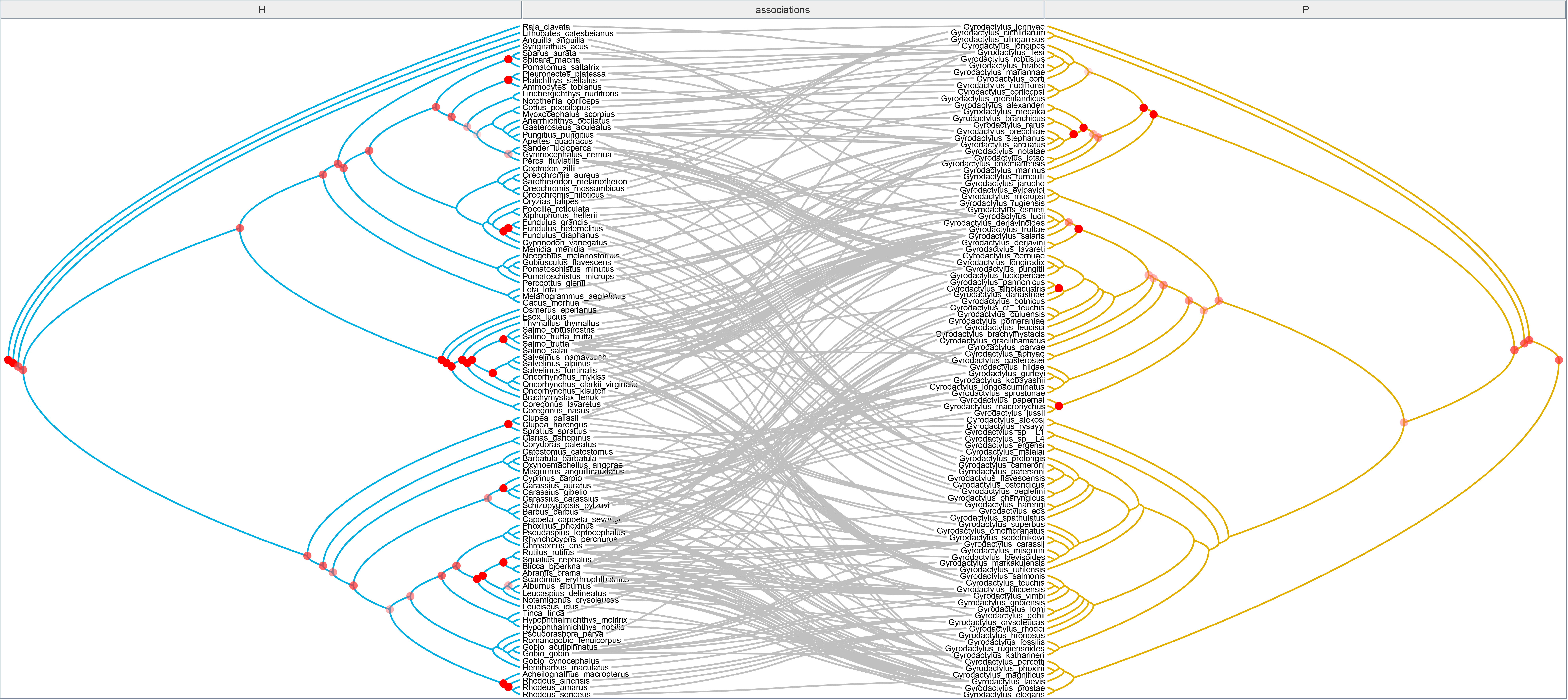


**Additional file 1: Figure S12:** the Treemap 3 tanglegram of the P18SHMITO dataset (BI topology). Red dots indicate nodes that exhibited significant congruence between host and parasite topologies. The intensity of the colour of the dot is positively correlated to the significance (p value) of congruence.


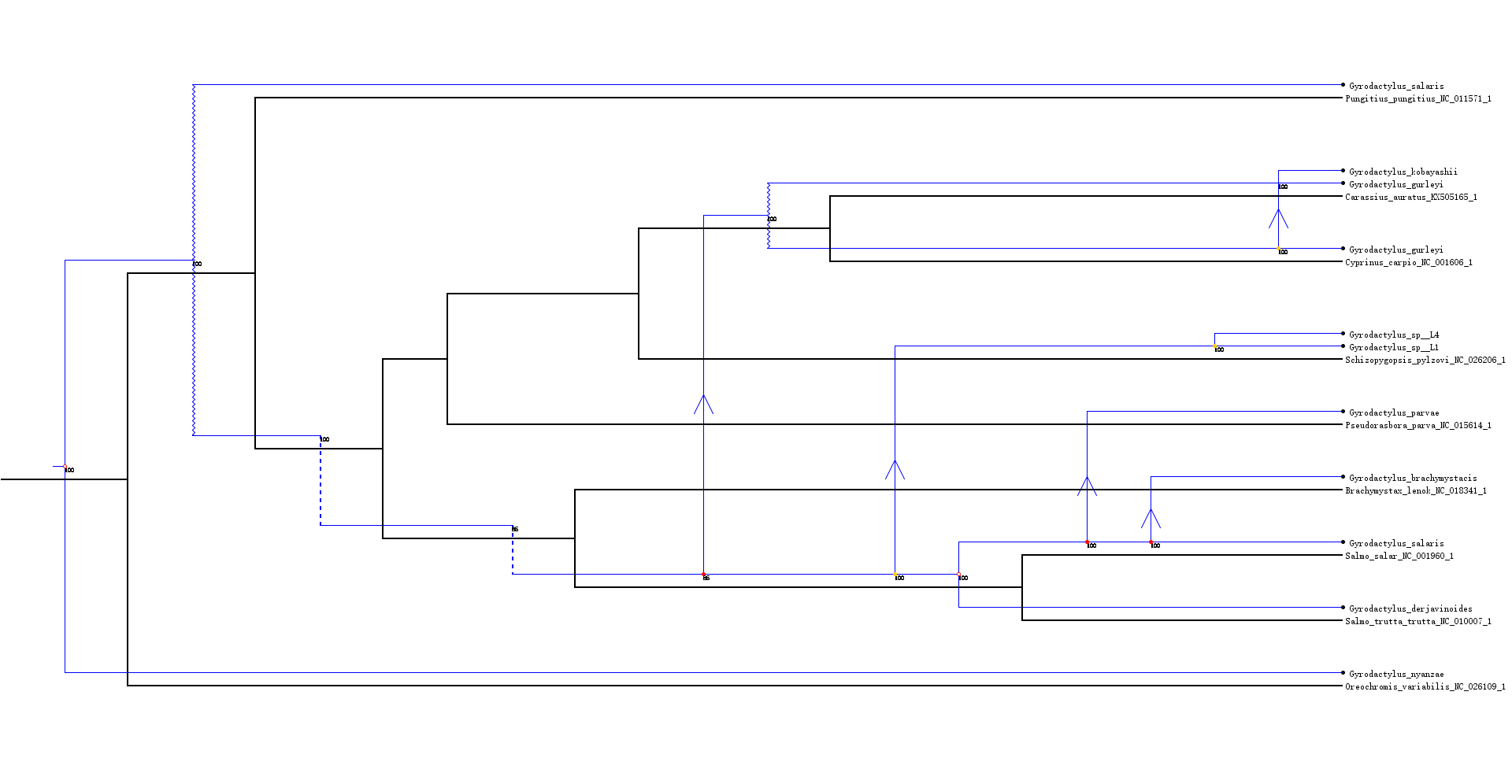


**Additional file 1: Figure S13:** the Jane 4 tanglegrams of the PHMITOS dataset (ML topology).


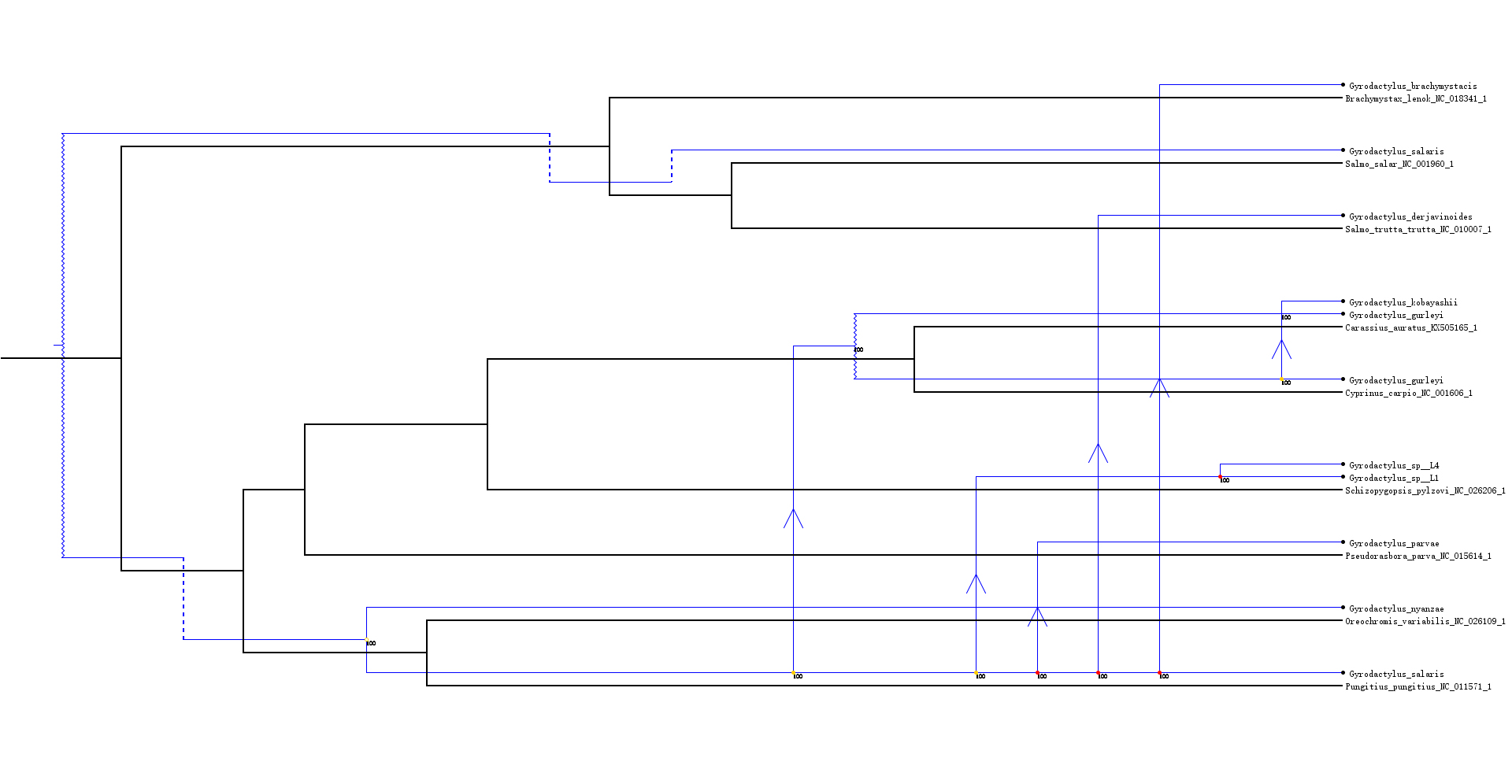


**Additional file 1: Figure S14:** the Jane 4 tanglegrams of the PHMITOS dataset (BI topology).


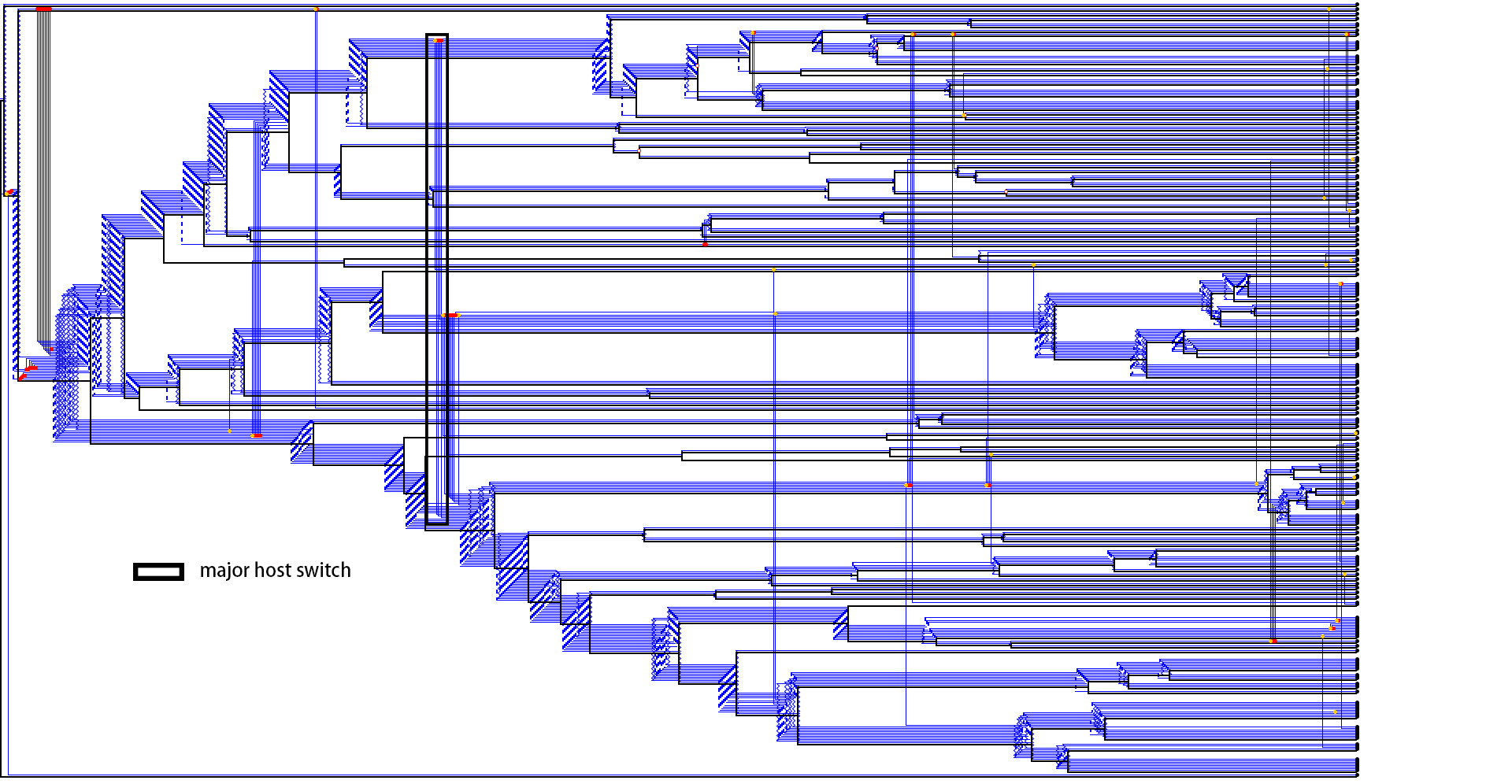


**Additional file 1: Figure S15:** the Jane 4 tanglegrams of the P18SHMITO dataset (ML topology).


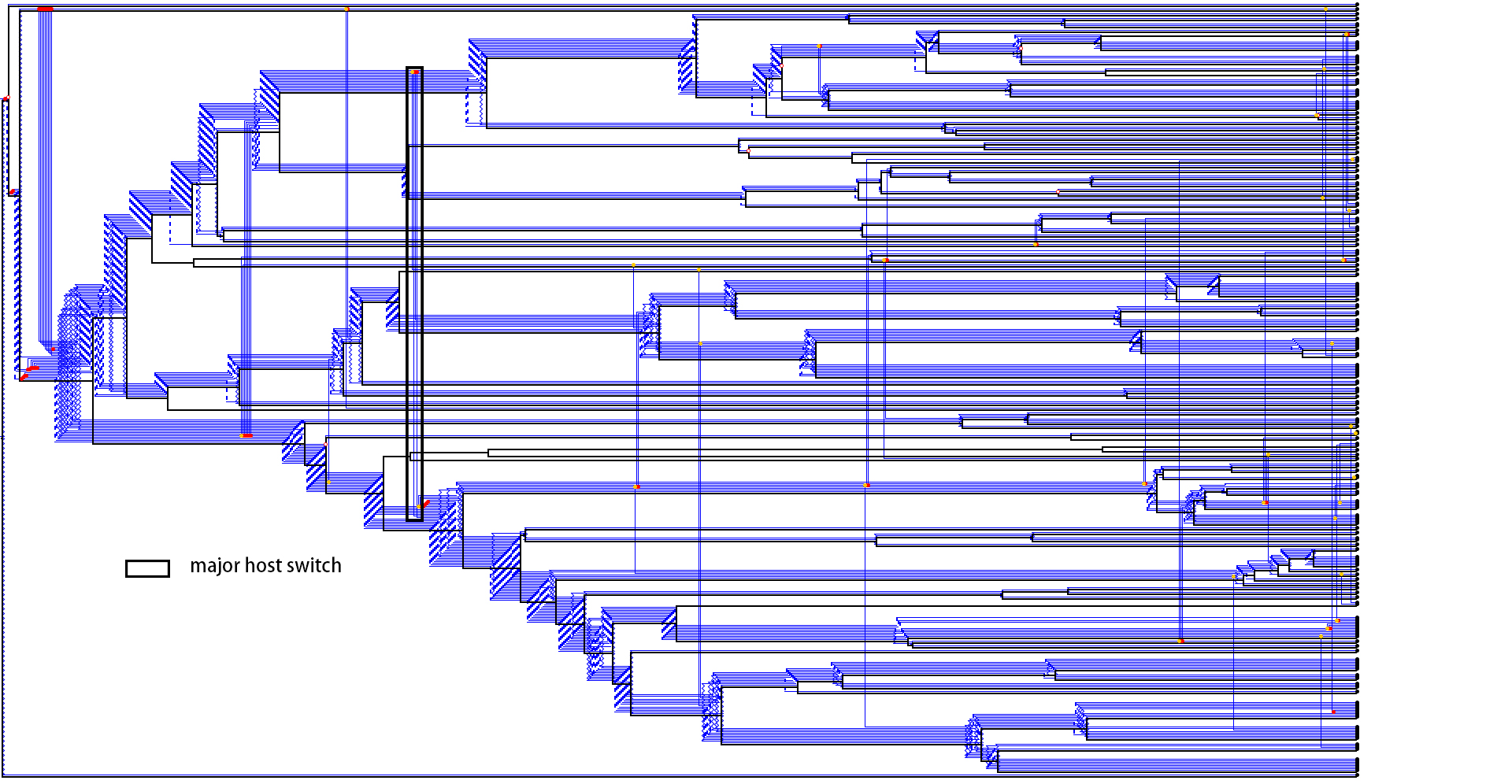


**Additional file 1: Figure S16:** the Jane 4 tanglegrams of the P18SHMITO dataset (BI topology).


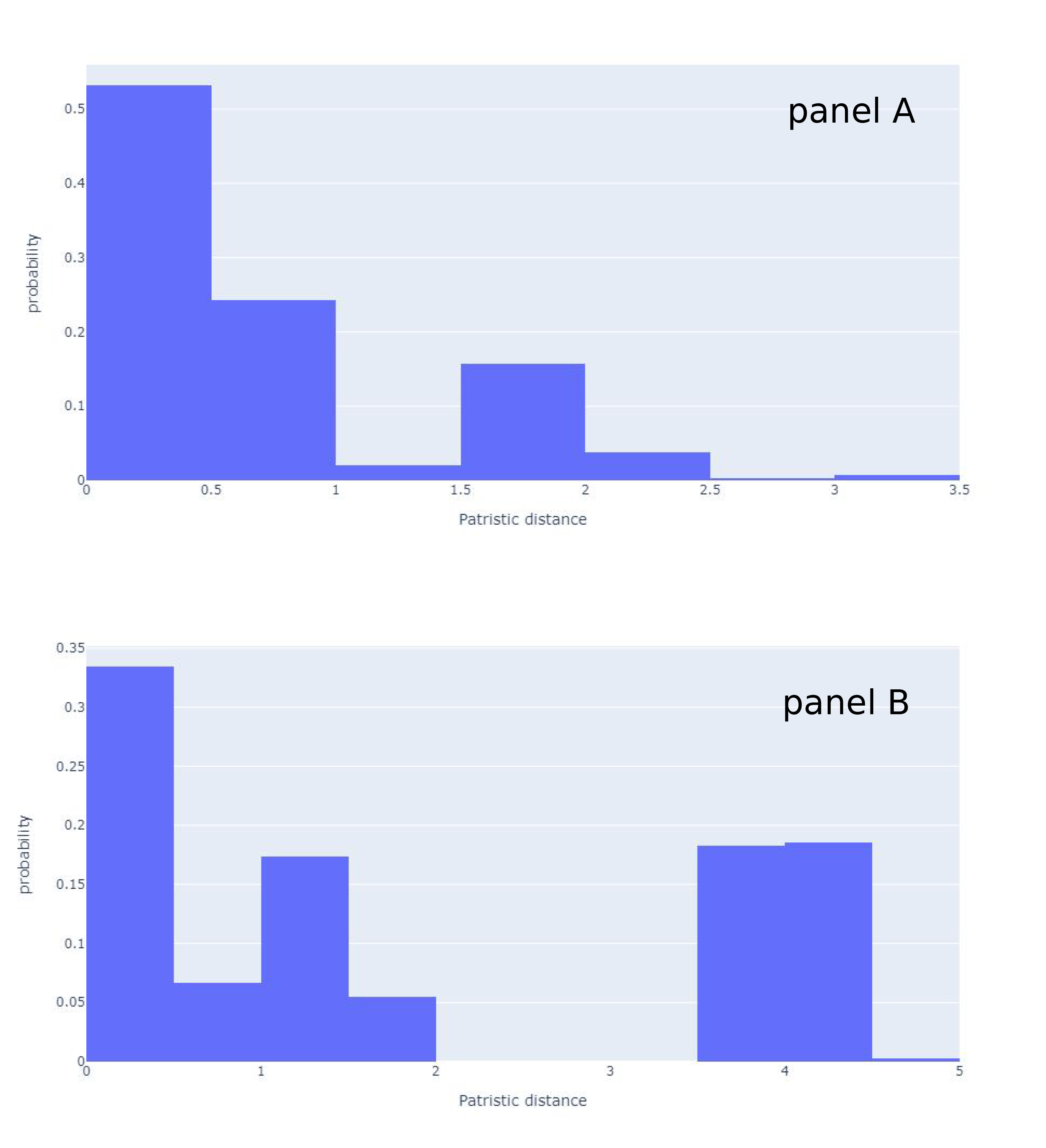


**Additional file 1: Figure S17:** panel A: The probability that a host pair with a gyrodactylid in common have a particular patristic phylogenetic distance, panel B: The probability that a gyrodactylid pair with a host in common have a particular patristic phylogenetic distance.


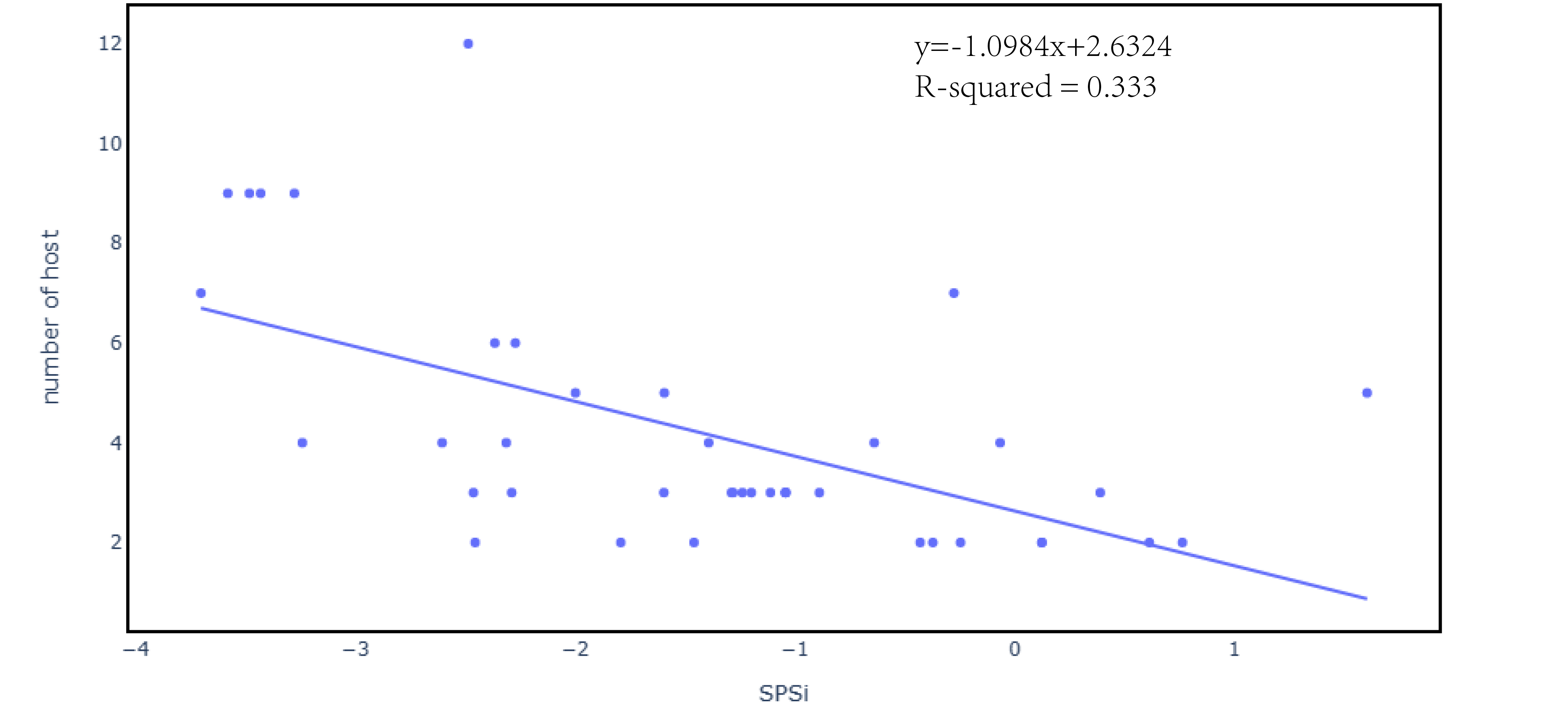


**Additional file 1: Figure S18:** The linear fit trendline of the basic host specificity (Y) and phylogenetic host specificity (X).
