# Supplementary material for: Geography, phylogeny and host switch drive the coevolution of parasitic Gyrodactylus flatworms and their hosts
Source: Parasit Vectors. 2024 Jan 30;17:42. doi: 10.1186/s13071-023-06111-6 (PMC10825989; doi:10.1186/s13071-023-06111-6)
Supplement: Supplementary file 2 — Additional file 2: Supplementary information. Supplementary material, methods, results, discussion and limitations of this study. [file 13071_2023_6111_MOESM2_ESM.docx]

Additional file **Information**

**Geography, phylogeny and host switch drive the coevolution of parasitic *Gyrodactylus* flatworms and their hosts**

Hong-Peng Lei^1^, Ivan Jakovlić^1^, Shun Zhou^2^, Xiang Liu^1^, Chuan Yan^1^, Xiao Jin^3^, Bo Wang^4^, Wen-Xiang Li^5^, Gui-Tang Wang^5^, Dong Zhang^1^*

^1^ State Key Laboratory of Herbage Improvement and Grassland Agro-ecosystems, and College of Ecology, Lanzhou University, 730000, Lanzhou, China.

^2^ Yangtze River Fisheries Research Institute, Chinese Academy of Fishery Sciences, Wuhan, 430223, China

^3^ College of Fishery, Guangdong Ocean University, Guangdong Provincial Key Laboratory of Aquatic Animal Disease Control and Healthy culture, Zhanjiang, China

^4^ Shapotou Desert Research and Experimental Station, Northwest Institute of Eco-Environment and Resources, Chinese Academy of Sciences, 320 Donggang West Road, Lanzhou 730000, People's Republic of China

^5^ Key Laboratory of Aquaculture Disease Control, Ministry of Agriculture, and State Key Laboratory of Freshwater Ecology and Biotechnology, Institute of Hydrobiology, Chinese Academy of Sciences, Wuhan, People’s Republic of China

* Correspondence: Dong Zhang, State Key Laboratory of Herbage Improvement and Grassland Agro-ecosystems, and College of Ecology, Lanzhou University, 730000, Lanzhou, China; Phone: +86-18717167887; Email: [dongzhang0725@gmail.com](mailto:dongzhang0725@gmail.com)

Additional file **material and methods**

**Sampling and sample identification**

Multiple *Gyrodactylus* sp. L1 and *Gyrodactylus* sp. L4 specimens were obtained from the fins of a freshwater fish species from the family Cyprinidae, *Schizopygopsis pylzovi,* caught by ourselves using traditional fishing tools in the Yellow River in Lanzhou city, Gansu Province (36.1709°N, 103.4683°E). Parasites were identified morphologically under a light microscope according to Shinn et al. [1]. All sampled parasites were first washed in 0.6% saline and then stored in 100% ethanol at 4 °C.

**DNA extraction, amplification and sequencing**

As these parasites are very small, to ensure a sufficient amount of DNA for amplification and sequencing, we used two kinds of genomic DNA: mixture DNA extracted from dozens of specimens and individual DNA extracted from a single specimen as described previously [2]. The ‘mixture DNA’ was extracted using the TIANamp MicroDNA Kit, whereas the ‘individual DNA’ was extracted using the standard phenol-chloroform method. DNA extraction was conducted on 12^th^ Jan 2022. Mixture DNA was used to amplify the whole mitogenome using degenerate primer pairs matching the generally conserved regions of mitochondrial genes. Specific primers were then designed according to these sequences to amplify and sequence the mitogenome. PCR mixture contained 10 µl 2 × PCR buffer, 0.6 µl of each primer, 0.4 µl rTaq polymerase (250 U, Takara), 1 µl of DNA template, and ddH_2_O up to 20 µl. Amplification conditions: 98 °C 2 min, 40 cycles at 98 °C 10 s, 48 - 60 °C 15 s, 68 °C 1 min/kb, and finally 68 °C 10 min. The PCR products were sequenced bidirectionally on an ABI 3730 automatic sequencer (Sanger method). Obtained fragments were checked via BLAST, and all sequencing chromatograms were inspected for double peaks or any other signs of the existence of two different sequences (to address the potential intraspecific sequence variation present in the mixture DNA). Then we used the ‘individual DNA’ and long-range PCR to verify the obtained mitogenomic sequence.

**Sequence annotation and analyses**

Both mitogenomes were assembled and annotated following the procedure described before (Zhang et al.,2019; Zhang et al.,2017)[3]. Briefly, after assembling them with the help of DNAstar v7.1 [4], MITOS was used to roughly annotate the mitogenome [5]. Boundaries of protein-coding genes (PCGs) and rRNA genes were manually fine-tuned via alignment with homologs. tRNAs were identified by combining the results of MITOS and ARWEN [6]. Genetic distances (identity) among mitogenomic sequences were computed with the ‘DistanceCalculator’ function in Biopython [7] using the ‘identity’ model.

# Additional file results

**Characteristic of the two gyrodactylids mitogenome**

The mitochondrial genomes of the two gyrodactylids were compared and the results are shown in supplementary table 1. The length of the complete mitochondrial genome of *Gyrodactylus sp.* L1 (OR031076) is 15447 bp and the complete mitochondrial genome of *Gyrodactylus sp.* L4 (OR031077) is 14900 bp (supplementary figure S1). Both genomes contain the standard 36 flatworm mitochondrial genes: 12 protein-coding genes (PCGs, atp8 not found), 22 tRNA genes and 2 rRNA genes.

Additional file 1: **Table S1. The annotated mitochondrial genome of *Gyrodactylus sp*. L1 and *Gyrodactylus sp*. L4 sequenced in this study.**

| Gene | Position | | Size | Intergenic nucleotides | Codon | | Identity |
| --- | --- | --- | --- | --- | --- | --- | --- |
|  | From | To |  |  | Start | Stop |  |
| *Gyrodactylus sp.* L1/*Gyrodactylus sp.* L4 | | | | | | | |
| *cytb* | 1/1 | 1074/1074 | 1074/1074 |  | ATG/ATG | TAA/TAA | 81.1 |
| *nad4L* | 1074/1074 | 1320/1320 | 247/247 | -1/-1 | ATG/ATG | T/T | 76.92 |
| *nad4* | 1295/1295 | 2500/2503 | 1206/1209 | -26/-26 | ATG/ATG | TAA/TAA | 68.23 |
| *trnF* | 2504/2507 | 2568/2572 | 65/66 | 3/3 |  |  | 89.39 |
| NCR | 2569/2573 | 3720/3341 | 1152/769 |  |  |  | 51.34 |
| *atp6* | 3721/3342 | 4233/3854 | 513/513 |  | ATG/ATG | TAA/TAG | 77.58 |
| *nad2* | 4236/3864 | 5093/4721 | 858/858 | 2/9 | ATG/ATG | TAG/TAA | 68.76 |
| *trnV* | 5103/4733 | 5163/4797 | 61/65 | 9/11 |  |  | 78.46 |
| *trnA* | 5167/4798 | 5235/4867 | 69/70 | 3/ |  |  | 91.43 |
| *trnD* | 5237/44868 | 5302/4933 | 66/66 | 1/ |  |  | 92.42 |
| *nad1* | 5303/4934 | 6190/5821 | 888/888 |  | ATG/ATG | TAG/TAG | 77.82 |
| *trnN* | 6190/5821 | 6255/5888 | 66/68 | -1/-1 |  |  | 88.24 |
| *trnP* | 6256/5889 | 6323/5955 | 68/67 |  |  |  | 80.88 |
| *trnI* | 6322/5952 | 6386/6016 | 65/65 | -2/-4 |  |  | 95.38 |
| *trnK* | 6388/6021 | 6452/6087 | 65/67 | 1/4 |  |  | 83.58 |
| *nad3* | 6456/6090 | 6803/6443 | 348/354 | 3/2 | ATG/ATG | TAG/TAG | 75.42 |
| *trnS1* | 6803/6443 | 6861/6501 | 59/59 | -1/-1 |  |  | 98.31 |
| *trnW* | 6873/6508 | 6939/6573 | 67/66 | 11/6 |  |  | 86.57 |
| *cox1* | 6944/6578 | 8491/8125 | 1548/1548 | 4/4 | ATG/ATG | TAA/TAA | 83.46 |
| *trnT* | 8504/8143 | 8578/8211 | 75/69 | 12/17 |  |  | 82.67 |
| *rrnL* | 8579/8212 | 9532/9159 | 954/948 |  |  |  | 84.97 |
| *trnC* | 9533/9160 | 9592/9219 | 60/60 |  |  |  | 70 |
| *rrnS* | 9593/9220 | 10292/9923 | 700/704 |  |  |  | 83.99 |
| *cox2* | 10293/9924 | 10874/10505 | 582/582 |  | ATG/ATG | TAA/TAG | 82.82 |
| NCR2 | 10875/10506 | 10991/10617 | 117/112 |  |  |  | 76.92 |
| *trnE* | 10992/10618 | 11058/10688 | 67/71 |  |  |  | 79.17 |
| *nad6* | 11062/10692 | 11544/11174 | 483/483 | 3/3 | ATG/ATG | TAA/TAA | 74.12 |
| *trnY* | 11546/11176 | 11611/11242 | 66/67 | 1/1 |  |  | 91.04 |
| *trnL1* | 11614/11246 | 11679/11311 | 66/66 | 2/3 |  |  | 92.42 |
| *trnQ* | 11683/11312 | 11743/11374 | 61/63 | 3/ |  |  | 90.48 |
| *trnM* | 11742/11373 | 11806/11437 | 65/65 | -2/-2 |  |  | 90.77 |
| NCR3 | 11807/11438 | 12903/12364 | 1097/927 |  |  |  | 56.77 |
| *trnS2* | 12904/12365 | 12963/12424 | 60/60 |  |  |  | 86.67 |
| *trnL2* | 12969/12428 | 13036/12495 | 68/68 | 5/3 |  |  | 86.76 |
| *trnR* | 13041/12501 | 13111/12569 | 71/69 | 4/5 |  |  | 81.69 |
| *nad5* | 13111/12569 | 14661/14116 | 1551/1548 | -1/-1 | ATG/ATG | TAA/TAA | 65.05 |
| *trnG* | 14673/14124 | 14737/14191 | 65/68 | 11/7 |  |  | 82.35 |
| *cox3* | 14741/14195 | 15379/14833 | 639/639 | 3/3 | ATG/ATG | TAA/TAA | 77.78 |
| *trnH* | 15380/14834 | 15444/14857 | 65/64 |  |  |  | 96.92 |

**Phylogenetic analyses**

***PHMITOS dataset***

ML and BI analyses of both datasets produced identical phylograms, with high bootstrap values (supplementary figure S2 and S3). As we did not have a suitable lineage to root the tree with, we used *Gyrodactylus nyanzae*, a species resolved as highly divergent from other *Gyrodactylus* species among nine included gyrodactylids in the study of Vanhove et al. [8]. The topology produced in our analysis was consistent with the above results of Vanhove et al. [8]. The newly sequenced *Gyrodactylus sp.* L1 and *Gyrodactylus sp.* L4 were sister lineages, but despite their close phylogenetic relationship, the *cox1* similarity value of only 83.5% confirms that they belong to different species.

***P18SHMITO dataset***

To determine the basal *Gyrodactylus* radiation, first we rooted the tree with *Paragyractylus variegatus* as the outgroup. In this topology, the clade comprising *Gyrodactylus laevis, Gyrodactylus pecotti, Gyrodactylus magnificus, Gyrodactylus phoxini, Gyrodactylus elegans* and *Gyrodactylus prostae* was the basal radiation within the *Gyrodactylus* clade, so we rooted the phylogenetic tree using the entire clade and used this tree in subsequent analyses (supplementary figure S4). ML and BI analyses of the parasite P18SHMITO dataset produced different phylograms (supplementary figure S5 and S6). The support values of many nodes in both phylograms were low, which indicates that the *18S+ITS* dataset did not contain sufficient phylogenetic signal*.* ML and BI analyses of the host P18SHMITO dataset produced similar phylograms with high support values (supplementary figure S7 and S8). The order Cypriniformes was monophyletic in both analyses, which was crucial for inferring inter-order host switches of gyrodactylids.

# Additional file discussion

**Coevolutionary events of gyrodactylids and their fish hosts**

Regarding the host switch-related events, the Jane 4 algorithm does not distinguish between “duplication & host switch” and ordinary host switch events. In more detail, duplication & host switch describes the process wherein a parasite first speciates within the same host (a form of sympatric speciation), and then one of the two species switches onto a different host. However, the Jane 4 algorithm does not consider the scenario wherein a parasite first switches to a different host, followed by speciation, which may also be common in nature. To address this shortcoming, we further divided the host switch-related events into two types: 1. both parasite speciation and host switch occurred (S host switch, which includes the duplication & host switch event in Jane 4), 2. parasite switched to a new host with no speciation (NS host switch).

The large number of failure to diverge events are probably related to the peculiar contact transmission of gyrodactylids. In more detail, if speciation of the host species is accompanied by geographic isolation, gyrodactylid parasites will also become split into two isolated populations, and eventually putatively also bifurcate into two species. However, if the host undergoes sympatric speciation, where the two new species continue to inhabit the same geographic habitat, their gyrodactylid parasites may have the opportunity to mate with each other.

The likelihood of gyrodactylids with high host specificity parasitize only on hosts with specific traits in loss events would be very low, unless there was a major disruptive event for the host, such as whole genome duplication, which might cause abrupt and major morphological and physiological changes. Besides, geographic isolation also needs to be considered. As fish hosts differentiate due to allopatric speciation, the population of their gyrodactylid parasites also experiences geographical isolation. However, parasites may be unable to adapt to the new environment and become extinct. Indeed, there are many reported cases where a gyrodactylid parasite failed to infect a fish species closely related to its primary host [9]. Our analyses support the previous proposition that, with the divergence of hosts, it is likely that one of the host lineages will manage to escape its parasites [10].

**Host specificity**

Host specificity, or the extent to which a parasite can exploit different host species, is a parameter fundamental for understanding host-parasite dynamics. Parasites with high host specificity parasitize only a single host species or a limited number of closely related species, while parasites with low host specificity are capable of using a diverse range of species as hosts. Ecologically, host-specificity reflects the magnitude of the parasite niche, and evolutionarily, it reflects the historical links of parasites to hosts.

Loss and failure to diverge events would have an impact on the number of hosts infected by gyrodactylids (i.e. basic host specificity). We also found that a large number of loss and failure to diverge events occurred on each ancestral node of host fishes with multiple parasites (Jane 4 results). As discussed earlier, whereas the loss events are relatively closely related to allopatric speciation, failure to diverge events are relatively closely related to sympatric speciation. In general, the geographical factor is expected to play an extremely important role in the occurrence of host fish with multiple parasites.

After removing gyrodactylids with only one host, the number of host species was significantly negatively associated (correlation coefficient = -0.58 and p value = 7.7E-05) with the SPS_i_ value (supplementary figure S18), whereas the two parameters were positively correlated when the full dataset was used (correlation coefficient = 0.45 and p value = 1.52E-06). This was probably due to some host-rich gyrodactylids exhibiting small SPS_i_ values (such as *Gyrodactylus laevis*, -3.58). This result indicated that gyrodactylids with multiple hosts tend to infect closely related hosts, which may be caused by failure to diverge events. These results also emphasize the importance of using SPSi to measure the host specificity of parasites, as the basic host specificity (host number) can often underestimate or overestimate the true host specificity. Even when it is accurate, the number of host species used by a parasite does not offer a sufficient resolution for a deep understanding of its evolution and ecological implications. However, some parasites with only two hosts also had positive SPS_i_ values (such as *Gyrodactylus pungitii* and *Gyrodactylus robustus*), which indicates the presence of major host switch events. Furthermore, among parasites sharing fish pairs in the last five patristic distance bins of supplementary figure S17 (panel A), 30.77 % had positive SPS_i_ (phylogenetic host specificity) values, indicating low host specificity. More than half (62.5%) of the fish pairs mentioned above were parasitized by two gyrodactylids: *Gyrodactylus salaris* and *Gyrodactylus arcuatus*. These two gyrodactylids have low basic host specificity (12 and 7 host species respectively) [9]and the phylogenetic distance between their hosts is quite large (average phylogenetic distance = 1.02 and 1.71 respectively), which caused high SPS_i_ values (3.82 and 4.81 respectively).

**Host switch**

As discussed above, gyrodactylids have different degrees of host specificity, which may be associated with their host-switching potential. In general, gyrodactylids can relatively easily transiently disperse to a new host species [11], but successful permanent colonization events are very rare for species with high host specificity [9]. However, in gyrodactylid species with lower host specificity, if successful permanent colonizations of new hosts (fish species) are commonly followed by speciation, speciation by host-switching might be an important speciation mode [9]. There is evidence of host switching in the coevolutionary history of monogeneans and their hosts from studies at the level of sister species [12] and among more basal branches in the monogenean phylogeny [13]. Zietara et al. [14] argued that ecological radiations of *Gyrodactylus* (diversification triggered by ecological factors) on phylogenetically distant fish species were attributable to host switch.

The highest probability of sharing the same host was observed for the most closely related gyrodactylids. Sympatric speciation of gyrodactylids was reported in *G. cf. Micropsi* and *G. cf. Micropsi* 1, two mutually closest relatives found on the same host species [15]. Intriguingly, we found a bimodal distribution of patristic distances of parasite pairs that shared the same fish host (supplementary figure S17, panel B). In other words, the probability that phylogenetically distant gyrodactylids pairs shared the same fish was also comparatively high (the total of 37.1% in the last three bins; panel B of supplementary figure S17 and table S2). For example, *Gyrodactylus arcuatus* and *Gyrodactylus carassii*, which both infect *Blicca bjoerkna*; and *Gyrodactylus arcuatus* and *Gyrodactylus flavescensis*, both of which infect *Gobiusculus flavescens*.

As we discussed earlier, major host switch could greatly enrich the diversity of gyrodactylids. However, the sampling of gyrodactylids on non-Cypriniformes hosts in our dataset was less detailed than sampling on Cypriniformes, as we did not have molecular data for some non-Cypriniformes hosts, so the proportion of gyrodactylids parasitizing non-Cypriniformes is likely to be underestimated. Finally, we found a few major host switch events that lead from the ancestral non-Cypriniformes hosts back to Cypriniformes hosts in more recent evolutionary history, which may also underestimate the estimates of major host switch events.

In summary, the strong colonizing capability, contact transmission, and epidemic spread of gyrodactylids facilitate occasional host switches, in rare cases even comprising phylogenetically distant hosts, which we named “major host switches”. Such rare major host switch events that occurred between the ancestral Cypriniformes and non-Cypriniformes have provided important opportunities for the diversification of gyrodactylids on non-Cypriniformes hosts. Therefore, despite the rarity of successful major host switches, they have major macroevolutionary importance. These results support the proposition that speciation by host-switching is expected to be an important speciation mode for gyrodactylids [9], and indicate that major host-switch events may be a major driver underlying the exceptional speciosity of *Gyrodactylus*.

**Limitations**

The *18S rRNA* exists in all eukaryotes and comprises both highly conserved and variable regions. The variable regions can be used to study relationships between closely related species and even within species, whereas conserved regions can be used to study evolutionary relationships between higher taxonomic levels [16]. As a non-coding region, the *ITS* evolves under lesser purifying selection pressures, so it evolves comparatively fast. The unstable topology and low support values in the *18S+ITS* phylogenies of gyrodactylids indicate that these sequences contained too weak a phylogenetic signal to completely resolve the phylogeny*.* Compared to *18S+ITS* molecular markers, the phylogenetic topology inferred from the mitochondrial genomes herein was very stable and had much higher support values. However, compared to the *18S+ITS* data, the availability of mitochondrial genome data is far lower. Another potential source of noise in our analyses stems from inconsistent criteria for species identification and associated taxonomic artefacts. Furthermore, there might be misidentified species in public databases, as some studies resolved *Gyrodactylus* as paraphyletic [8], and the information on host ranges obtained from GyroDb may be incomplete, or even putatively erroneous in some cases. Finally, some of the loss events identified in this study may also be artefacts caused by incomplete sampling and identification of fish parasites in the wild [17].

Gyrodactylids with only one host make up 23.3% of the parasite dataset, but their SPS_i_ values could not be calculated. As deleting them would represent a major reduction of the dataset, and they are expected to have the lowest phylogenetic specificity, we manually set their SPS_i_ values to match the lowest SPS_i_ value in the rest of the dataset (-4.7). This was a relatively crude shortcut, but it made a large impact on our results, as the inclusion of these species completely changed some of the conclusions (see the discussion of scatterplot results).

As we used two different datasets which had different resolutions, they produced incongruent results, especially among Treemap 3, Jane 4 and Parafit algorithms. For example, the significance of congruence between host and parasite subtrees in the parasite tree significantly differed in Treemap 3 results for these two datasets, and the dominant types of coevolutionary events completely differed in Jane 4 results. Finally, although the coevolutionary relationship was significant in Parafit analyses of both datasets, they produced very different p values.

The unstable topology and low support values in the *18S+ITS* phylogenies of gyrodactylids indicate that these sequences contained too weak a phylogenetic signal to completely resolve the phylogeny. The resolution was further weakened by several species being represented by only partial sequences. Thus, multilocus mitogenomic or genomic datasets carrying a large amount of phylogenetic signal may be needed to conduct a high-resolution gyrodactylids-hosts coevolutionary study. Although we did apply such a dataset, the resolution was severely weakened by poor lineage sampling. The dataset that we used for network analysis was also weakened by a relatively small number of species. Aside from the fact that geographic distribution data may be imprecise for multiple fish species, a major shortcoming was that we were forced to assume that the geographic distribution of parasites fully overlaps with that of their hosts due to the lack of geographic distribution data information for gyrodactylids. This, of course, may not fully correspond to the actual geographic distribution of parasites and may overinflate the importance of geography in downstream analyses. However, previous studies have shown that patterns in parasite species richness closely tracked host species richness across space, and that this relationship is robust to deviations from the assumption that parasites match the distribution of their hosts [18]. In addition, to assess the relative impacts of phylogeny and geography on host-parasite interactions, it would be necessary to sample all hosts and all (gyrodactylid) parasites at a single location.

**References**

1. Shinn PA, Hansen H, Olstad K, Bachmann L, Bakke AT. The use of morphometric characters to discriminate specimens of laboratory-reared and wild populations of Gyrodactylus salaris and G. thymalli (Monogenea). Folia Parasitologica. 2004;51 2:239-52. <https://folia.paru.cas.cz/artkey/fol-200402-0016.php>

<http://dx.doi.org/10.14411/fp.2004.029>.

2. Zhang D, Li WX, Zou H, Wu SG, Li M, Jakovlić I, et al. Mitochondrial genomes and 28S rDNA contradict the proposed obsoletion of the order Tetraonchidea (Platyhelminthes: Monogenea). Int J Biol Macromol. 2020;143:891-901; doi: 10.1016/j.ijbiomac.2019.09.150. <http://europepmc.org/abstract/MED/31726130>

https://doi.org/10.1016/j.ijbiomac.2019.09.150.

3. Zhang D, Zou H, Wu SG, Li M, Jakovlić I, Zhang J, et al. Sequencing of the complete mitochondrial genome of a fish-parasitic flatworm Paratetraonchoides inermis (Platyhelminthes: Monogenea): tRNA gene arrangement reshuffling and implications for phylogeny. Parasites & Vectors. 2017;10 1:1-12.

4. Burland TG. DNASTAR’s Lasergene sequence analysis software. Bioinformatics methods and protocols: Springer; 2000. p. 71-91.

5. Bernt M, Donath A, Jühling F, Externbrink F, Florentz C, Fritzsch G, et al. MITOS: Improved de novo metazoan mitochondrial genome annotation. Molecular Phylogenetics and Evolution. 2013;69 2:313-9; doi: <https://doi.org/10.1016/j.ympev.2012.08.023>. <https://www.sciencedirect.com/science/article/pii/S1055790312003326>.

6. Laslett D, Canbäck B. ARWEN: a program to detect tRNA genes in metazoan mitochondrial nucleotide sequences. Bioinformatics. 2008;24 2:172-5; doi: 10.1093/bioinformatics/btm573. <https://doi.org/10.1093/bioinformatics/btm573>.

7. Cock PJA, Antao T, Chang JT, Chapman BA, Cox CJ, Dalke A, et al. Biopython: freely available Python tools for computational molecular biology and bioinformatics. Bioinformatics. 2009;25 11:1422-3; doi: 10.1093/bioinformatics/btp163. <https://doi.org/10.1093/bioinformatics/btp163>.

8. Vanhove MPM, Briscoe AG, Jorissen MWP, Littlewood DTJ, Huyse T. The first next-generation sequencing approach to the mitochondrial phylogeny of African monogenean parasites (Platyhelminthes: Gyrodactylidae and Dactylogyridae). BMC Genomics. 2018;19 1:520; doi: 10.1186/s12864-018-4893-5. <https://doi.org/10.1186/s12864-018-4893-5>.

9. Bakke TA, Harris PD, Cable J. Host specificity dynamics: observations on gyrodactylid monogeneans. International Journal for Parasitology. 2002;32 3:281-308; doi: <https://doi.org/10.1016/S0020-7519(01)00331-9>. <https://www.sciencedirect.com/science/article/pii/S0020751901003319>.

10. Hamerlinck G, Hulbert D, Hood G, Smith J, Forbes A. Histories of host shifts and cospeciation among free‐living parasitoids of Rhagoletis flies. Journal of evolutionary biology. 2016;29 9:1766-79.

11. Bakke TA, Cable J, Harris PD. The Biology of Gyrodactylid Monogeneans: The “Russian-Doll Killers”. In: Baker JR, Muller R, Rollinson D, editors. Advances in Parasitology. vol. 64: Academic Press; 2007. p. 161-460.

12. Guégan J-F, Agnèse JF. Parasite evolutionary events inferred from host phylogeny : the case of Labeo species (teleostei, cyprinidae) and their dactylogyrid parasites (monogenea, dactylogyridae). Canadian Journal of Zoology. 1991;69:595-603.

13. Boeger WA, Kritsky DC. Coevolution of the Monogenoidea (Platyhelminthes) based on a revised hypothesis of parasite phylogeny. International Journal for Parasitology. 1997;27 12:1495-511; doi: <https://doi.org/10.1016/S0020-7519(97)00140-9>. <https://www.sciencedirect.com/science/article/pii/S0020751997001409>.

14. Zietara MS, Lumme J. SPECIATION BY HOST SWITCH AND ADAPTIVE RADIATION IN A FISH PARASITE GENUS GYRODACTYLUS (MONOGENEA, GYRODACTYLIDAE). Evolution. 2002;56 12:2445-58; doi: <https://doi.org/10.1111/j.0014-3820.2002.tb00170.x>. <https://doi.org/10.1111/j.0014-3820.2002.tb00170.x>.

15. Huyse T, Audenaert V, Volckaert FAM. Speciation and host–parasite relationships in the parasite genus Gyrodactylus (Monogenea, Platyhelminthes) infecting gobies of the genus Pomatoschistus (Gobiidae, Teleostei). International Journal for Parasitology. 2003;33 14:1679-89; doi: <https://doi.org/10.1016/S0020-7519(03)00253-4>. <https://www.sciencedirect.com/science/article/pii/S0020751903002534>.

16. Hadziavdic K, Lekang K, Lanzen A, Jonassen I, Thompson EM, Troedsson C. Characterization of the 18S rRNA gene for designing universal eukaryote specific primers. J PloS one. 2014;9 2:e87624.

17. Charleston MA, Perkins SL. Traversing the tangle: Algorithms and applications for cophylogenetic studies. Journal of Biomedical Informatics. 2006;39 1:62-71; doi: <https://doi.org/10.1016/j.jbi.2005.08.006>. <https://www.sciencedirect.com/science/article/pii/S1532046405000791>.

18. Harris NC, Dunn RR. Using host associations to predict spatial patterns in the species richness of the parasites of North American carnivores. Ecology Letters. 2010;13 11:1411-8; doi: <https://doi.org/10.1111/j.1461-0248.2010.01527.x>. <https://doi.org/10.1111/j.1461-0248.2010.01527.x>.
